# Blockchain-enabled data governance for privacy-preserved sharing of confidential data

**DOI:** 10.7717/peerj-cs.2581

**Published:** 2024-12-20

**Authors:** Jingchi Zhang, Anwitaman Datta

**Affiliations:** 1 College of Computing and Data Science, Nanyang Technological University, Singapore, Singapore; 2 De Montfort University Leicester, Leicester, United Kingdom

**Keywords:** Attribute-based encryption, Blockchain, Data sharing, Multi-authority, Privacy

## Abstract

In traditional cloud storage systems, users benefit from the convenience of data accessibility but face significant risks related to security. Ciphertext-policy attribute-based encryption (CP-ABE) schemes are employed to achieve fine-grained access control in cloud services to ensure confidentiality while maintaining data-sharing capabilities. However, existing approaches are impaired by two critical issues: illegal authorization and privacy leakage. Despite extensive discussions in the literature on interoperability, performance, scalability, and stability, the security of ABE-based cloud storage and data-sharing systems against adversaries—particularly those involving adaptively corrupt attribute authorities gaining unauthorized access to users’ data—has not been sufficiently explored. Notably, few existing works even address security in the presence of adversaries, raising concerns about the practicality of these systems in real-world scenarios where malicious behavior is a genuine threat. Another pressing issue is privacy leakage, where sensitive user information, such as medical histories in healthcare use cases, embedded within the access policies, may be exposed to all users. This problem is exacerbated in ABE schemes that integrate blockchain technology for enhanced decentralization and interoperability, as using a public ledger shared across multiple users can further compromise privacy. To address these, we propose an enhanced blockchain-based data governance system that employs blockchain technology and attribute-based encryption to prevent illegal authorization and privacy leakage. Our novel ABE encryption system supports multi-authority use cases while hiding access policy and ensuring identity privacy, which also protects data sharing against corrupt authorities. Utilizing the Advanced Encryption Standard (AES) for data encryption, our system is optimized for real-world efficiency. Notably, the encrypted data is stored in a decentralized storage system, like the InterPlanetary File System (IPFS), which does not rely on any centralized service provider and can, therefore, be leveraged to achieve resilience against single-point failures. With the integration of smart contracts and multi-authority attribute-based encryption, coupled with blockchain’s inherent transparency and traceability, our system realizes a balanced solution for fine-grained access control with preserved privacy, further fortifying against credential misuse. Besides the system design, we also present security proofs to demonstrate the robustness of the proposed system.

## Introduction

Notwithstanding the many advantages of cloud computing, which have led to its widespread adoption and continuous growth, it also presents certain risks that prompt the exploration of alternative architectures. In particular, due to the inherent centralization of cloud services, they can become a single point of failure. This presents issues regarding service availability, censorship, and end-user privacy concerns. These challenges are further exacerbated by potential insider attacks and the service provider’s own agency. For instance, Apple’s decision in 2021 to roll out a Child Sexual Abuse Material detection technology by scanning images stored in its iCloud service led to numerous collateral privacy concerns and criticisms (Mitchell, 2022). Even though the original plan was eventually scuppered because of strong public backlash, the fundamental vulnerability of such centralized systems, which are subject to privacy violations or censorship, remains. (Portions of this text were previously published as part of a preprint (Zhang & Datta, 2023)).

To address some of these issues inherent in centralized cloud storage, many encryption schemes such as AES, RSA, proxy re-encryption, identity-based encryption, and attribute-based encryption (ABE) have been used to secure data confidentiality (Sudha & Monica, 2012; Alowolodu et al., 2013; Yan, Rong & Zhao, 2009; Yang et al., 2020). However, some encryption schemes may not be amenable to a wide variety of common data-sharing use cases.

Consider a complex surgical procedure that requires collaboration among specialized experts from around the world. These experts need access to the patient’s electronic health records (EHRs) to plan the operation and ensure all necessary equipment and preparations are in place. Therefore, the patient (data owner, *DO*) must securely share their private EHRs with doctors, hospitals, and other relevant institutions (data users, *DU*s) involved in the surgery. The primary challenge is ensuring that the EHRs are shared with the appropriate professionals on a need-to-know basis without exposing sensitive patient information, while also supporting flexible accessibility so that new DUs can access the EHRs without significant additional effort. Relying on a single trusted entity for data sharing can introduce risks, especially if that entity is compromised, and no single doctor or hospital typically has contact with all the required specialists.

To address this, a reliable and decentralized data-sharing system is essential. This system could publicly advertise the medical requirements to attract qualified experts worldwide while authorizing specific vetted individuals and institutions to access the patient’s EHRs. In this context, a blockchain-based data-sharing system is preferable (Wang, Zhang & Zhang, 2018; Gao et al., 2020; Qin et al., 2021). Blockchain’s key features—immutability, transparency, and decentralized control—offer a secure environment for sharing sensitive medical information. By integrating advanced encryption techniques, the system ensures that only authorized parties with the correct access privileges can view and interact with the EHRs, maintaining patient privacy and safeguarding data integrity.

In a traditional public-key encryption (PKE) system like RSA or an Identity-Based Encryption (IBE) scheme, the patient must re-encrypt the EHR using the doctor or institution’s public key or generate a new identity-based ciphertext. These access control options are limited in flexibility and scalability, as the *DO* may have to take additional steps on demand to ensure that the encrypted content is accessible to a new *DU*. In contrast, Ciphertext-Policy Attribute-based Encryption (CP-ABE) schemes offer a more flexible approach. Successful decryption can be carried out only if a user’s attribute set satisfies the access policy embedded in the ciphertext. Therefore, regardless of whether the number of users is predetermined, new *DU*s can access the EHRs without requiring significant additional effort from the *DO*.

However, current CP-ABE schemes may introduce privacy issues. The embedded policy in the encrypted EHR could reveal the identity of potential *DU*s, leading to the unintended disclosure of the patient’s information (Wu, Xu & Zhu, 2023). This issue is exacerbated when such information is uploaded on-chain to leverage the benefits of trustable and immutable logging (Wu, Xu & Zhu, 2023), whether using a public ledger like the Ethereum network or a private ledger like Hyperledger Fabric. For instance, records indicating visits to specialized hospitals or interactions with an insurance company may be used to infer a patient’s condition (Gao et al., 2020). Unauthorized entities, such as pharmaceutical companies that target patients with advertisements, could exploit this information. Similarly, other hospitals or insurance companies might use this data for marketing or discriminate pricing. It is thus crucial to hide the access policy to protect patient privacy.

Another practical concern is the risk of illegal authorization, where a corrupt authority might issue attribute keys to unauthorized *DU*s (Hei et al., 2021). Revisiting the use case above, companies interested in accessing patients’ information could bribe an attribute authority to obtain decryption keys. Alternatively, a company acting as an attribute authority might introduce a ‘backdoor’ during the setup phase, allowing future unauthorized access. Consequently, ensuring the security of blockchain-based data-sharing systems against corrupt authorities is also essential.

### Motivations

In this subsection, we elaborate on prior ABE schemes’ limitations and practical challenges, highlighting the need for our work.

#### Decentralization

In traditional cloud services, the data owner (*DO*) typically uploads ciphertext to a cloud storage server, which means the user may take the risk of assuming that the cloud storage server will provide reliable service to ensure the availability of the uploaded data (Wang, Zhang & Zhang, 2018). However, this reliance inherently introduces a single point of failure. Another drawback of centralized cloud services arises when integrating CP-ABE for improved flexibility and scalability: it relies on intermediary entities like a trusted third party (TTP) and a central authority (CA) to perform operations such as attribute key distribution, policy verification, and data retrieval honestly. These operations are critical for ensuring the security and trustworthiness of data access control, but the reliance on centralized entities poses significant risks.

To better safeguard data availability and ensure the reliability and traceability of operations, it is crucial to design decentralized alternatives for trust mechanisms and enforce traceability throughout the access control system (Wang, Zhang & Zhang, 2018). Consider the application scenario described above: if the potential data users (*DUs*) are not determined in advance, and the purpose of data sharing also includes attracting public attention, such as coordinating a challenging surgery or managing world-level tasks, a blockchain-based solution is inherently advantageous. Blockchain’s decentralized nature allows for broader participation and enables the system to support complex, multi-faceted applications that require secure and transparent access control mechanisms.

A blockchain-based access control system with CP-ABE has the potential to address these issues effectively. By leveraging blockchain, we can eliminate the need for a centralized TTP to control the data. Each node in the network maintains a distributed ledger that tracks a growing list of transactions, which are verified and confirmed by consensus mechanisms before being recorded. The integrity of transactions can be secured by hashing, Merkle trees, time stamping, and incentive mechanisms. This hybrid approach enhances resilience against single-point failures and the misuse of credentials by ensuring that no single entity has complete control over the access control system. Additionally, the decentralized nature of blockchain inherently supports transparency and traceability, preventing any individual authority or entity from acting maliciously without detection (Gao et al., 2020; Hei et al., 2021; Wu et al., 2019).

#### Practicality

Several blockchain-based access control systems have been proposed since the emergence of public blockchain systems (Nakamoto, 2008) and the advent of Attributed-based Encryption (Sahai & Waters, 2005). Some efforts leverage the immutable public ledger to build a transaction-based access control system for secure data sharing (Maesa, Mori & Ricci, 2017; Ouaddah, Abou Elkalam & Ait Ouahman, 2016; Wang, Zhang & Zhang, 2018). In contrast, others leverage the self-executing smart contracts to establish a smart contract-based access control system for flexibility and traceability (Qin et al., 2021; Hei et al., 2021; Wu, Xu & Zhu, 2023). However, just employing blockchain technology and CP-ABE encryption for an access control system is inadequate for several practical purposes, such as cross-domain data sharing and privacy leakage.

On the one hand, information is not always shared inside a single domain or organization. For example, driver’s licenses and university registration information may be managed by separate entities. If one central authority is responsible for attribute management and key distribution like the proposed system (Kaur, Rani & Kalra, 2024), it also has trust issues, as discussed above. Therefore, multi-authority attribute-based encryption (MA-ABE), originally proposed by Chase (2007), is used to solve the access problem involving attributes belonging to various authorities. This scheme permits any number of independent authorities to distribute secret keys, which the data owner later chooses to encrypt a message. However, this MA-ABE scheme also relies on a *CA* that issues seeds to each AA, giving the *CA* the capability to decrypt any ciphertext. To eliminate the need for a ‘super-power’ *CA* and achieve full decentralization, Lewko & Waters (2011) proposed a fully secure decentralized CP-ABE solution without requiring cooperation among multiple AAs.

Another issue is privacy concerns, which encompass both policy-hiding and receiver privacy. Since in the classic CP-ABE schemes, an access structure specified in terms of user attributes is explicitly transmitted alongside ciphertext, whoever accesses the ciphertext is also aware of the corresponding access policy. Therefore, multi-authority CP-ABE schemes (Chase, 2007; Lewko & Waters, 2011; Rouselakis & Waters, 2015) are still unsuitable for certain use cases since access policies contain sensitive information. This calls for mechanisms to hide access policies for CP-ABE systems. Additionally, *DU* needs to provide a full set of user attributes to each authority for an attribute key, inevitably compromising the key receiver’s privacy.

In pursuit of addressing these concerns, several CP-ABE schemes that feature policy-hiding have been proposed (Cui et al., 2018; Zhang, Zheng & Deng, 2018; Yang et al., 2018; Gao et al., 2020; Michalevsky & Joye, 2018; Zhang et al., 2021; Kaur, Rani & Kalra, 2024). Despite these efforts, they do not completely fulfill various practical requirements. Some of them (Cui et al., 2018; Zhang, Zheng & Deng, 2018; Gao et al., 2020; Kaur, Rani & Kalra, 2024) only support a single central authority. Schemes such those from Yang et al. (2018) and Zhang et al. (2021) are prone to the leakage of *DU*’s confidential attribute information during the key generation or encryption process. Michalevsky & Joye (2018) introduced a fully policy-hiding decentralized CP-ABE scheme, which protects attribute information attached to the access policy and even addresses the issue of receiver privacy.

#### Security

Beyond the basic security goals of access control systems, such as data confidentiality, ABE schemes need to address another security issue: collusion between users. Specifically, even if data users (*DU*s) collude by sharing their attribute keys, they should not be able to decrypt ciphertexts unless each of their issued attribute keys **individually** satisfies the access policy (Bethencourt, Sahai & Waters, 2007). Additionally, different types of ABE-based systems address varying security concerns, such as accountability, which is essential in accountable ABE (Zhang et al., 2020b).

In the context of multi-authority or decentralized ABE, the security goal of collusion resistance is further complicated by the possibility of keys being issued by different authorities. More importantly, the corruption of some but not all authorities should not compromise the confidentiality of the system’s data (Lewko & Waters, 2011). However, many existing ABE-based solutions either fail to adequately address security in the presence of adversaries, as seen in the works like (Xue et al., 2017; Wang, Zhang & Zhang, 2018; Wu, Xu & Zhu, 2023), or define security goals for their application scenarios with noticeable omissions, as found in schemes (Gao et al., 2020; Nasiraee & Ashouri-Talouki, 2020; Porwal & Mittal, 2020). In general, the adversarial models in these works are often overly idealized and do not reflect real-world scenarios.

The issue of corrupted attribute authorities is a widely discussed security problem in most existing ABE-based systems that support multi-authority environments (Yang et al., 2018; Qin et al., 2021; Zhang et al., 2021; Zhao et al., 2022). However, these systems are typically proven secure only against static corruption of authorities, where enquiring about corrupt authorities are made at the beginning of the game (Chen et al., 2023). This implies that the set of corrupted authorities must be fixed, and authority keys must be requested upfront, limiting the adversary’s ability to adaptively change its attack strategy. Moreover, these schemes assume that all attribute authorities must join the system simultaneously, an assumption that is impractical in real-world deployments.

We further identify that several ABE schemes realized through inner-product predicate encryption (Michalevsky & Joye, 2018; Tseng & Gao, 2022) are vulnerable to **rogue-key attacks** under the fully adaptive security model, where an adversary can corrupt authorities at any point in time. In such an attack, a malicious attribute authority (*AA*) can generate and register an aggregate public key based on public information from other honest authorities. This rogue key can then be used to decrypt ciphertexts without possessing the necessary attribute keys to satisfy the access policy. Furthermore, works such as Zhang et al. (2020a) and Agrawal, Goyal & Tomida (2021), which build upon the scheme in Michalevsky & Joye (2018), may also inherit this vulnerability.

Another potential issue in the **setup** phase of the MA-ABE scheme, which has not drawn as much attention as corrupt authorities, is the reliance on a central or trusted authority for the generation of the global public key. For instance, in works such as Hei et al. (2021), only the central authority is involved in this global setup process. This assumption introduces the risk of a single point of failure and overlooks the potential for adversaries to introduce a “backdoor” during setup, which could be exploited later to carry out more harmful attacks on ciphertexts. We also identify a potential leakage of sensitive information in the scheme proposed by Michalevsky & Joye (2018) if a trusted setup is not employed for the generation of the global public key.

As a result, the challenge of securely storing user data, enabling efficient data sharing, and managing multi-authority scenarios while concurrently maintaining a balance of decentralization, traceability, privacy, security, and efficiency constitutes a complex problem that requires innovative solutions.

### Contributions

In this paper, we propose a multi-party CP-ABE-based storage outsourcing system that uses blockchain technology to address decentralization, practicality, and security problems. Our solution achieves fine-grained access control by allowing data owners to define precise access policies based on user attributes while ensuring user anonymity by concealing both access policies and user identities during data access. Additionally, it is resilient against rogue-key attacks under the fully adaptive corruption assumption introduced in the work Datta, Komargodski & Waters (2023), ensuring stronger security compared to other Inner Product Predicate Encryption (IPPE)-based schemes, even in the presence of adaptively corrupted authorities.

The core contributions of this work are as follows:

**Table 1:** Summary of access control system using attribute-based encryption (Part I).

**Approach**	**Authority**	**Policy**	**Universe**	**Policy-hiding**	**Receiver-hiding**	**Access control**	**Storage**
Wang, Zhang & Zhang (2018)	Single	AND	Small	No	No	Smart contract	IPFS
Cui et al. (2018)	Single	LSSS	Large	Partially	No	CSP	CSP
Zhang, Zheng & Deng (2018)	Single	LSSS	Large	Partially	No	CSP	CSP
Yang et al. (2018)	Multiple	LSSS	Small	No	Yes	CSP	CSP
Gao et al. (2020)	Single	AND	Large	Fully	No	Smart contract	CSP
Qin et al. (2021)	Multiple	LSSS	Small	No	No	CSP	CSP
Zhao et al. (2022)	Multiple	LSSS	Large	Fully	No	CSP	CSP
Tseng & Gao (2022)	Multiple	AND	Large	No	No	CSP	CSP
This work	Multiple	AND	Small	Fully	Yes	Smart contract	IPFS

• **Capability gap and vulnerability analysis of the state-of-the-art:** We examine several widely discussed ABE schemes that support multi-authority and privacy-preserving properties and select the scheme presented in Michalevsky & Joye (2018) as the most suitable for real-world scenarios to build our data-sharing system upon. We then closely analyze this scheme under a more realistic security model, **fully adaptive security**, and identify that it is vulnerable to a rogue-key attack, where a malicious *AA* can decrypt ciphertext without possessing the necessary attribute keys required to satisfy the policy. Furthermore, the scheme is exposed to a potential risk where an adversary might infer sensitive information from the published ciphertext due to poorly chosen public parameters. These vulnerabilities are thoroughly analyzed in “Attack” and “Vulnerability”, respectively.
• **Rogue-key attack resilient protocol design:** To counteract the rogue-key attack and alleviate some potential risks, we modify the algorithms of **Setup** and **Auth Setup** in Michalevsky & Joye (2018) as described in Definition 5. Firstly, we introduce a multi-party protocol inspired by Bowe, Gabizon & Green (2018) for public key generation, which is detailed in **Trusted Setup** of “Trusted Setup”. Secondly, we impose a prerequisite for each *AA* to prove the knowledge of published information during the process of **Auth Setup**. This is elaborated in “Authority Setup”. We further demonstrate that our enhanced system successfully mitigates the aforementioned security concerns, as outlined in “Proof of security of our approach” and “Proof of security with our approach”.
• **System architecture for blockchain integration:** In order to incorporate transparency and decentralization, we integrate blockchain technologies such as smart contracts and content addressing, alongside multi-authority attribute-based encryption. An overview of the system architecture is presented in “System Overview”. This hybrid approach enhances the practicality and security of the system, which makes it resilient against single-point failures and misuse of credentials. Given that transparency and traceability are inherent attributes of blockchain, a blockchain-enabled ABE system realizes a balanced solution for data sharing while simultaneously preserving privacy.
• **Comprehensive comparison with related works:** We provide a comprehensive comparison of existing ABE-based data-sharing systems in terms of decentralization, privacy, and security, as discussed in “Related Work” and summarized in Table 1. To position our proposed work relative to existing solutions regarding efficiency, we evaluate computational complexity in “Asymptotic Comparisons” and present the experimental results comparing two closely related IPPE-based schemes with our enhanced construction, along with analysis in “Experimental Result”.

Overall, we propose a secure, privacy-preserving data governance system based on blockchain technology and an improved decentralized policy-hiding CP-ABE scheme with receiver privacy. Using a combination of ABE and the Advanced Encryption Standard (AES) makes the system practical. The special ABE encryption scheme is capable of handling multi-authority use cases while protecting identity privacy and ABE’s policy. The adoption of AES helps assure the confidentiality of user data, which is furthermore stored in a decentralized storage system, specifically the InterPlanetary File System (IPFS), which does not rely on a central service provider, thus avoiding a single point of failure.

### Organization

The rest of the paper is structured as follows: “Related Work” contains related work that reviews traditional Attribute-based Encryption schemes and conducts an analysis of some recent solutions for access control systems with ABE technology, elaborated in Table 1. “Preliminary” summarizes the preliminaries that the techniques developed in this paper build upon. The proposed system protocol is overviewed in “System Overview” and discussed in depth in “System Design”. “Security Analysis” contains systematic security analysis, while “Performance Analysis” provides a comparative study of our system against related works. Finally, our conclusions and future plans are presented in “Concluding Remarks”.

## Related Work

ABE was first introduced by Sahai and Waters in 2005 (Sahai & Waters, 2005). Subsequently, numerous proposals for a single-authority ABE system (Goyal et al., 2006; Bethencourt, Sahai & Waters, 2007) have been put forth. In these systems, the data owner (*DO*) encrypts data and employs a boolean formula over a set of attributes to restrict access. If the data user (*DU*) possesses the secret keys issued by a central authority (*CA*) that satisfy the boolean formula attached to the ciphertext, *DU* can retrieve the original data. However, these single-authority ABE systems (Sahai & Waters, 2005; Goyal et al., 2006; Bethencourt, Sahai & Waters, 2007) encounter constraints such as performance bottlenecks and key escrow issues.

Therefore, Zhang et al. (2014) proposed an enhanced ABE scheme, which alleviates the performance bottleneck issue by reducing the computation cost and ciphertext length. It has been further explored in Wang, Zhang & Zhang (2018) to create a framework that integrates decentralized storage, smart contract, and CP-ABE techniques to achieve fine-grained access control.

Another concern with the single-authority ABE system is key escrow, where *CA* issues all the attribute secret keys, thereby gaining the ability to decrypt each ciphertext generated by data owners. To address this issue, Chase & Chow (2009) introduced a multi-authority attribute-based (MA-ABE) scheme without the need for CA. Lewko & Waters (2011) further developed this multi-authority scheme in their work allowing any authority to join or leave the system independently. Based on it, Qin et al. (2021) designed a blockchain-based multi-authority access control scheme to address performance and single-point failure issues.

In an effort to extend the usability of ABE schemes, Nishide et al. presented a desirable property, hidden access policy, in Nishide, Yoneyama & Ohta (2008). This approach protects sensitive attribute values while leaving attribute names public, denoted as partially hiding. Since then, multiple enhanced schemes (Lai, Deng & Li, 2011; Cui et al., 2018; Zhang, Zheng & Deng, 2018; Gao et al., 2020; Xu et al., 2023b) have been proposed. To support a wide variety of access structures, a fully secure policy-hiding ABE was proposed in Lai, Deng & Li (2011). Gao et al. (2020) used the optimized scheme of Lai, Deng & Li (2011) to build a blockchain-based access control system that achieves trustworthy access while maintaining the privacy of policy and attributes. To improve the expressiveness of the access policy, a partially hidden ABE scheme under the Linear Secret Sharing Scheme (LSSS) policy was proposed in Cui et al. (2018). Zhang, Zheng & Deng (2018) proposed a privacy-aware access control system, denoted as PASH, which supports a large universe ABE scheme with partially hidden ABE. There are several similar approaches providing policy-hiding as well as ensuring accountability for key abuse, for example, Li’s work (Li et al., 2022) based on large universe ABE construction (Rouselakis & Waters, 2013) and the scheme of Wu et al. (2019) based on attribute bloom filter (ABF) (Dong, Chen & Wen, 2013). We also note that there is a longer list of desired features, such as keyword-searchable technology in ABE schemes or decentralized settings, as explored in works like (Wang, Zhang & Zhang, 2018; Xu et al., 2023b; Xu et al., 2023a).

Nevertheless, most of the aforementioned schemes either neglect the attribute of policy-hiding or exist as single-authority ABE systems. This gap is addressed by multi-authority attribute-based encryption schemes with a hidden access policy (Zhong et al., 2016; Belguith et al., 2018; Michalevsky & Joye, 2018; Zhao et al., 2022). The MA-ABE scheme featuring policy-hiding was initially introduced by Zhong et al. (2016), and subsequently improved by Belguith et al. (2018) that significantly diminishes computational cost by delegating the decryption work to a semi-trusted cloud server.

In addition to the above, there are a few other proposals Yang et al. (2018), Zhao et al. (2022) in this area that, unfortunately, give rise to additional issues. For instance, a system developed by Yang et al. (2018) keeps the user’s identity private from the attribute authority (*AA*) if they are not in the same domain. Yet, this approach creates a new privacy issue that users might request *AA*s within the domain to ask secret attribute keys from other *AA*s on their behalf, implying that an *AA* could potentially possess a complete set of a *DU*’s secret keys. Zhao et al. (2022) presented a data sharing scheme that adopts the access policy of linear secret sharing scheme (LSSS) and supports the MA-ABE scheme with policy-hiding to achieve privacy-preserving functionality. However, this system is vulnerable to user key abuse due to its dependence on a single central authority for key generation.

In 2018, Michalevsky and Joye introduced the first practical multi-authority attribute-based encryption (MA-ABE) scheme with the policy-hiding property (Michalevsky & Joye, 2018), realized through Inner-Product Predicate Encryption (IPPE). This scheme provides a security proof in the random oracle model, against static corruption of authorities, where the list of corrupted authorities is fixed at the beginning of the security game. The scheme also supports various access policy types, including conjunctions, disjunctions, and threshold policies. Additionally, Michalevsky & Joye (2018) addressed the issue of receiver privacy through the use of vector commitment. Notably, this scheme is one of the few that achieves fully hiding CP-ABE, ensuring that no attribute information is leaked with the access policies. Fully hiding CP-ABE can only be achieved indirectly *via* IPE or through threshold policies (Zhang et al., 2020b). Consequently, it is reasonable to build a data-sharing system based on this scheme, with appropriate modifications, to support real-world applications such as electronic health records or financial records.

However, the scheme of Michalevsky & Joye (2018) has its limitations, including support for only fixed-size attributes and authorities, and the need for coordination among authorities during the setup phase. More critically, we demonstrate that the scheme is vulnerable to a rogue-key attack in the presence of adaptively corrupted authorities, where corruption queries can be made at any point in time. This notion is later formalized as fully adaptive security by the work of Datta, Komargodski & Waters (2023) at EuroCrypt 2023. In real-world decentralized settings, it is realistic to expect that some authorities may join the data-sharing system later, due to factors such as network delays. Thus, the assumption that all authorities join simultaneously and that the list of corrupted authorities is fixed at the beginning of the security game does not reflect the dynamic and unpredictable nature of real-world scenarios. In practice, attackers may adaptively change their strategies based on the information available at any given time. The fully adaptive security model perfectly captures this situation and should be used to evaluate the security of proposed systems, ensuring they are practical enough to support real-world use cases. Specifically, in a rogue-key attack, a compromised authority may decrypt ciphertexts even without possessing the required attribute keys.

In Table 1, we compare and position our blockchain-enabled data-sharing system with existing works Wang, Zhang & Zhang (2018), Cui et al. (2018), Zhang, Zheng & Deng (2018), Yang et al. (2018), Gao et al. (2020), Qin et al. (2021), Zhao et al. (2022), Tseng & Gao (2022) that are closely related to ours with regard to flexibility, scalability, privacy, and decentralization across the following assessment criteria:

1. Attribute authority: Whether the authorities involved in CP-ABE schemes are divided into single thus central authority or multi-authority.
2. Policy: LSSS which supports AND gate, OR gate, and threshold gate *versus* only AND.
3. Attribute universe: We define the complete set of supported attributes as an attribute universe and only take into account two types of the universe: the large universe and the small universe. In large universe ABE, the attribute universe size has no effect on the size of the system’s public key.
4. Privacy: There are two aspects of privacy involved in CP-ABE schemes: policy-hiding and receiver-hiding. For the policy-hiding scheme, the CP-ABE system is available in two forms: fully hidden and partially hidden. The former means that none of the attributes can be revealed from the access policies, whereas the latter refers to only hiding sensitive attribute values in the access policies. For the receiver-hiding scheme, it prevents any *AA*s from learning the full set of attributes the receiver (*i.e.,* the *DU*) possesses, hence relieving the *DU* from disclosing them while requesting attribute keys.
5. Storage: From a technical perspective, traditional cloud service provider (CSP) and decentralized storage systems such as IPFS, Storj, and Sia, are two distinct popular solutions for data storage and sharing. CSPs may take advantage of their comprehensive control over data, but end users are exposed to the risks of a single point of failure, privacy violation, and censorship.
6. Access control: We indicate whether access control enforcement is through a smart contract and thus logically decentralized or by a cloud service provider and thus logically centralized.

From Table 1, we observe that very few schemes (Li et al., 2022; Yang et al., 2018; Zhao et al., 2022) achieve fine-grained access control and support multi-authority with privacy-preserved characteristics, such as policy-hiding and receiver privacy. However, they all rely on a trusted third party (TTP) or cloud service provider (CSP) to offer centralized storage and access control management and are thus susceptible to the inherent vulnerabilities of such centralized systems in terms of privacy issues. In contrast, our proposed scheme stands out by leveraging smart contracts for access control management and integrating with the IPFS network for storage to realize an architecture with completely decentralized data storage and governance.

## Preliminary

To initiate, we revisit certain foundational principles employed within our system. A summary of crucial notations utilized throughout the manuscript is provided in Table 2.

**Table 2:** Notation description.

**Notation**	**Description**
*p*	A prime number used for 𝔾_1_, 𝔾_2_, 𝔾_*T*_ and ℤ
𝔾_1_, 𝔾_2_	Two additive cyclic groups.
𝔾_*T*_	A multiplicative cyclic group.
ℤ_*p*_	A set of integers with order *p*
*λ*	A security parameter for the input size
$\mathcal{U}$	A set of attribute authorities in the universe
$\mathcal{X}$	A set of attributes in the universe
$\mathcal{S}$	A set of attributes possessed by each attribute authority
$\mathcal{R}$	A set of attributes possessed by data user
*n*	The number of attribute authorities
*l*	The number of supported attributes
*GID*	A Data User’s Global identifier
*k*	The parameter for the *k-lin* assumption, representing the linear independence of group elements.
*PP*	Public parameters for the use of Attribute-Based Encryption or Vector Commitment
*α*	A scalar used for generation of *PP*
*e*	A set of secret elements used for ** Trusted Setup** or ** Authority Setup**
*h*	A hash of committed elements in ** Trusted Setup** of ** Authority Setup**
*π*	A proof of knowledge for an element
***rp***_*s*_	A *s-pair* of the element *s* in group 𝔾_1_. The superscript 2 of ${\mathbi{rp}}_{s}^{2}$ represent *s-pair* elements in group 𝔾_2_
*L*	A list of *s-pair* consisting of all the committed group elements
(*PK*, *SK*)	A key pair which is used for ABE encryption
***X***, *τ*, *σ*	A set of secret elements in *SK*
***A***, ***U***	The secret exponents used in *PP* of ABE
*K*	A component of the attribute key for each individual attribute
*sk*	The consolidated secret key issued by an attribute authority. Given that an attribute authority can oversee multiple attributes, *sk* might comprise several *K* components
***x***	A policy vector
***v***	An attribute vector
***C***	A Vector Commitment associated with a specific Data User, derived from its attribute vector and global identifier
*m*	A special message used in Vector Commitment
*op*	An opening proof to reveal the Vector Commitment
*o*_*i*_, *o*_*i*,*j*_	The elements in *PP*_*VC*_ where *i*, *j* ∈ [*n*], *i* ≠ *j*
*z*	A secret exponent of group element *o*
***aux***	A collection of message *m*
*BPK*	A public key registered in a blockchain
*BSK*	A private key registered in a blockchain

### Bilinear mapping

Consider $\mathcal{G}$ as an algorithm that accepts a security parameter *λ* and constructs three multiplicative cyclic groups of prime order *p*: 𝔾_1_ = 〈*g*_1_〉, 𝔾_2_ = 〈*g*_2_〉, and 𝔾_*T*_. We introduce $\hat {e}$ as a bilinear map, with $\hat {e}:{\mathbb{G}}_{1}\times {\mathbb{G}}_{2}\rightarrow {\mathbb{G}}_{T}$. The bilinear map $\hat {e}$ has the following characteristics:

1. **Bilinearity**: for all *a*, *b* ∈ ℤ, $\hat {e}({g}_{1}^{a},{g}_{2}^{b})=\hat {e}({g}_{1},{g}_{2})^{ab}$.
2. **Non-degeneray**: $\hat {e}({g}_{1},{g}_{2})\not = 1$.
3. **Computability**: for all *a*, *b* ∈ ℤ, $\hat {e}({g}_{1}^{a},{g}_{2}^{b})$ can be efficiently computed.

### Auxiliary methods and definitions

We make an assumption of possessing an algorithm, denoted as COMMIT, which takes strings of arbitrary length as input and produces outputs as determined by a random oracle. While this assumption aids our security analysis, in practical implementations, we could use the BLAKE-2 hash function in place of COMMIT. For the inputs *h* that can not be mapped directly to integers, especially in the case of group elements, we represent them using byte-strings.

Additionally, we introduce several auxiliary methods to facilitate the verification procedure for certain special properties.

The following definitions and claims are first proposed in the work Bowe, Gabizon & Green (2018).

Definition 1 Given a bilinear mapping $\hat {e}:{\mathbb{G}}_{1}\setminus \{ 0\} \times {\mathbb{G}}_{2}\setminus \{ 0\} \rightarrow {\mathbb{G}}_{T}$, elements *A*, *B* ∈ 𝔾_1_∖{0} and *C*, *D* ∈ 𝔾_2_∖{0}. If $\hat {e}(A,D)=\hat {e}(B,C)$, we may use the term **SameRatio**((*A*, *B*), (*C*, *D*)) to represent this relation.

 
____________________________ 
Algorithm 1 Determin if two pairs (A,B) and (C,D) have certain relationship________________________ 
Require: A,B ∈ G1∖{0} and C,D ∈ G2∖{0} 
 1:  function SAMERATIO((A,B),(C,D)) 
  2:       if ˆ e (A,D) = ˆ e (B,C) then 
 3:            return true 
  4:       else 
 5:            return false 
  6:       end if 
 7:  end function___________________________________________________________________________________________

Definition 2 Given a bilinear mapping $\hat {e}:{\mathbb{G}}_{1}\setminus \{ 0\} \times {\mathbb{G}}_{2}\setminus \{ 0\} \rightarrow {\mathbb{G}}_{T}$, $s\in {\mathbb{Z}}_{p}^{\ast }$ and cyclic group of order *p*, an *s-pair* is a pair (*A*, *B*) such that *A*, *B* ∈ 𝔾_1_, or *A*, *B* ∈ 𝔾_2_; and *s*⋅*A* = *B*. For such an *s-pair* (*A*, *B*) in 𝔾_1_ or 𝔾_2_, we may represent it using the notation ***rp***_*s*_ or ${\mathbi{rp}}_{s}^{2}$ respectively.

Claim 1 ***SameRatio*** ((*A*, *B*), (*C*, *D*)) =* true if and only if there exists s such that* (*A*, *B*)*is an *s-pair* in 𝔾_1_ and (*C*, *D*) is an *s-pair* in 𝔾_2_.*

Finally, we can construct our special *s-pair* as follows.

Definition 3 Given a bilinear mapping $\hat {e}:{\mathbb{G}}_{1}\setminus \{ 0\} \times {\mathbb{G}}_{2}\setminus \{ 0\} \rightarrow {\mathbb{G}}_{T}$ and a matrix $s\in {\mathbb{Z}}_{p}^{l\times k}$, a special *s-pair* is a pair (*A*, *B*) such that $A,B\in {\mathbb{G}}_{1}^{l\times k}$ or $A,B\in {\mathbb{G}}_{2}^{l\times k}$; and

\begin{eqnarray*}B[i,j]=A[i,j]^{s[i,j]} \end{eqnarray*}

For such a special *s-pair* (*A*, *B*) in 𝔾_1_ or 𝔾_2_, we may also denote it as ***rp***_*s*_. Given that a vector can be considered a matrix with a single column, we can also use the notation ***rp***_*s*_ to represent an *s-pair* when $s\in {\mathbb{Z}}_{p}^{k}$.

### Assumptions

Given a bilinear mapping $\hat {e}:{\mathbb{G}}_{1}\setminus \{ 0\} \times {\mathbb{G}}_{2}\setminus \{ 0\} \rightarrow {\mathbb{G}}_{T}$ with associated generators {*g*_1_, *g*_2_, *g*_*T*_} and group order *p*, our work builds upon a variety of standard assumptions, which are detailed below.

Assumption 1 Symmetric External Diffie-Hellman (SXDH) assumption (Ballard et al., 2005). It is hard to distinguish ${\mathcal{D}}_{0}=({g}_{1},{g}_{2},{g}_{1}^{a},{g}_{1}^{b},{g}_{1}^{ab})$ from ${\mathcal{D}}_{1}=({g}_{1},{g}_{2},{g}_{1}^{a},{g}_{1}^{b},{g}_{1}^{c})$ where $a,b,c\leftarrow _{}^{\text{$}}{\mathbb{Z}}_{p}$. This also holds to the tuples ${\mathcal{D}}_{0}^{{^{\prime}}}=({g}_{1},{g}_{2},{g}_{2}^{a},{g}_{2}^{b},{g}_{2}^{ab})$ and ${\mathcal{D}}_{1}^{{^{\prime}}}=({g}_{1},{g}_{2},{g}_{2}^{a},{g}_{2}^{b},{g}_{2}^{c})$ in different group.

Assumption 2 K-Linear assumption (Boneh, Boyen & Shacham, 2004). It is hard to distinguish ${\mathcal{D}}_{0}=({g}_{1},{g}_{2},{g}_{1}^{{a}_{1}},{g}_{1}^{{a}_{2}},\ldots ,{g}_{1}^{{a}_{k}},{g}_{1}^{{a}_{1}{b}_{1}},{g}_{1}^{{a}_{2}{b}_{2}},$

$\ldots ,{g}_{1}^{{a}_{k}{b}_{k}},{g}_{1}^{{b}_{1}+{b}_{2}+\cdots +{b}_{k}})$ from ${\mathcal{D}}_{1}=({g}_{1},{g}_{2},{g}_{1}^{{a}_{1}},{g}_{1}^{{a}_{2}},\ldots ,{g}_{1}^{{a}_{k}},{g}_{1}^{{a}_{1}{b}_{1}},$

${g}_{1}^{{a}_{2}{b}_{2}},\ldots ,{g}_{1}^{{a}_{k}{b}_{k}},{g}_{1}^{c})$ where ${a}_{1},\ldots ,{a}_{k},{b}_{1},\ldots ,{b}_{k},c\leftarrow _{}^{\text{$}}{\mathbb{Z}}_{p}$. This also holds in the group 𝔾_2_.

The matrix ***A*** and vector ***a***^⊥^ are defined as:

(1) \begin{eqnarray*}\mathbi{A}= \left[ \begin{array}{@{}c@{}} \displaystyle \text{diag}({a}_{1},{a}_{2},\ldots ,{a}_{k})\\ \displaystyle {\mathbf{1}}^{\top } \end{array} \right] \end{eqnarray*}

(2) \begin{eqnarray*}{\mathbi{a}}^{\perp }={ \left( \begin{array}{@{}c@{}} \displaystyle {a}_{1}^{-1},{a}_{2}^{-1},\ldots ,{a}_{k}^{-1},-1 \end{array} \right) }^{\top }\end{eqnarray*}

where the *a*_*i*_ are sampled elements from ℤ_*p*_. These are constructed such that ***A***^⊺^***a***^⊥^ = 0.

Assumption 3 Special k-Linear assumption (Michalevsky & Joye, 2018). Given a randomly generated matrix $\mathbi{A}\in {\mathbb{Z}}_{p}^{(k+1)\times k}$ and a vector $\mathbi{s}\in {\mathbb{Z}}_{p}^{(k+1)}$, the tuples ${\mathcal{D}}_{0}=({g}_{1},{g}_{2},{g}_{1}^{\mathbi{A}},{g}_{1}^{\mathbi{As}})$ and ${\mathcal{D}}_{1}=({g}_{1},{g}_{2},{g}_{1}^{\mathbi{A}},{g}_{1}^{\mathbi{s}})$ are computationally indistinguishable by any pol-ynomial-time $\mathcal{A}$. The structure of matrix ***A*** is described in Eq. (1) and the vector ***a*** is derived from ***A*** as detailed in Eq. (2).

Assumption 4 Square Computational Diffie-Hellman assumption (Burmester, Desmedt & Seberry, 1998). Given (*g*, *g*^*a*^) for a random number *a* in a cyclic group 𝔾 of order *p*, a *PPT* algorithm $\mathcal{A}$ outputs *g*^*a*^2^^ with non-negligible probability.

Assumption 5 Knowledge of Coefficient assumption (Bowe, Gabizon & Miers, 2017). Given a string of arbitrary length *h*, and a uniformly chosen *C* ∈ 𝔾_2_ (independent of *h*), an efficient algorithm $\mathcal{A}$ exists that can randomly generate *B* ∈ 𝔾_1_ and *D* ∈ 𝔾_2_. Meanwhile, for the same inputs (*C*, *h*), there is an efficient deterministic algorithm $\mathcal{X}$ cable of extracting a scalar *b*. The probability that both are true: (1) $\mathcal{A}$ ‘succeeds’, meaning it satisfies the condition that SameRatio ((*g*_1_, *B*), (*C*, *D*))

(2) $\mathcal{X}$ ‘fails’, meaning $B\not = {g}_{1}^{b}$

is negligible.

### Proof of knowledge

We adopt the well-established Schnorr identification protocol (Schnorr, 1990), utilizing it as our Non-interactive Zero-knowledge (NIZK) proof. Provided with an *s-pair rp*_*s*_ = (*A*, *B* = *s*⋅*A*) and a string *h*, we establish NIZK ( Algorithm 2 ). This can serve as proof that the originator of the string *h* is aware of *s* in the *s-pair rp*_*s*_.

Furthermore, we define VerifyNIZK ( Algorithm 3 ), which checks the validity of the provided proof *π*.

 
________________________________________________________________________________________________________________ 
Algorithm 2 Construct a proof of knowledge of s_____________________________________________________ 
Require: rps is an s-pair 
Require: h is a string 
  1:  function NIZK(rps = (A,B),h) 
  2:       α  $ 
 ←− Z∗p 
  3:       R ← α ⋅ A 
 4:       c ← COMMIT(R||h) ∈ Z∗p 
  5:       u ← α + c ⋅ s 
 6:       return π = (R,u) 
  7:  end function___________________________________________________________________________________________    

 
________________________________________________________________________________________________________________ 
Algorithm 3 Verify a proof of knowledge of s_________________________________________________________ 
Require: rps is an s-pair 
Require: h is a string 
  1:  function VERIFYNIZK(rps = (A,B),π = (R,u),h) 
  2:       c ← COMMIT(R||h) ∈ Z∗p 
  3:       if u ⋅ A == R + c ⋅ B then 
 4:            return true 
  5:       else 
 6:            return false 
  7:       end if 
 8:  end function___________________________________________________________________________________________ 

### Vector commitment

We ensure our attribute-hiding property through the utilization of a Vector Commitment scheme, as described in Catalano & Fiore (2013). The summarized scheme is as follows:

Definition 4 This Vector Commitment system commits to an ordered sequence of attribute elements ***v*** = (*v*_1_, *v*_2_, …, *v*_*l*+1_) as commitment ***C***, then opens it in a certain position of ***v*** to a corresponding attribute authority (*AA*), and finally proves that only authorized value existed in the previously supplied commitment ***C***. The system normally consists of four algorithms:

• **Key generation** (1^*λ*^, *n*) → *PP*_*VC*_: This is a decentralized key generation (DKG) algorithm. It takes as input the security parameter *λ* and the number of attribute authorities, *n*, in the system, and outputs global public parameters *PP*_*VC*_ = {*g*_1_, *g*_2_, {*o*_*i*_}, {*o*_*i*,*j*_}} where *i*, *j* ∈ [*n*], *i* ≠ *j*. The element *o*_*i*_ is generated and published by *AA*_*i*_. Following that, the elements {*o*_*i*,*j*_} can be issued by each *AA*_*i*_ based on the shared {*o*_*i*_}.
• **Commitment** (***aux*** = {*m*_*i*_}_*i*∈[*n*]_) → ***C***: This algorithm is run by a data user (*DU*). It takes as input the message *m*_*i*_ generated based on the authorized attributes from *AA*_*i*_, *i* ∈ [*n*], and outputs the commitment ***C***.
• **Open** (*m*_*i*_, *i*, ***aux***) → *op*_*i*_: This algorithm is also run by a *DU*. It takes as input the auxiliary information ***aux*** and index *i*, and outputs the opening proof *op*_*i*_.
• **Verify** (***C***, *m*_*i*_, *i*, *op*_*i*_) → (1 or 0): The Verify algorithm is run by *AA*. It takes as inputs the commitment ***C***, message *m*_*i*_, index *i*, and opening proof *op*_*i*_, and outputs the result of the verification. It outputs 1 when it accepts the proof.

### Decentralized inner-product predicate encryption

Definition 5 A multi-authority attribute-based encryption with policy-hiding scheme (Michalevsky & Joye, 2018) consists of a tuple of probabilistic polynomial-time (PPT) algorithms, such that:

• **Setup** (1^*λ*^) → *PP*: It takes as input the security parameter *λ* and then outputs the public parameters *PP*.
• **Authority setup** (*PP*, *i*) → (*PK*_*i*_, *SK*_*i*_): It takes as input public parameter *PP* and authority index *i*, and outputs a pair of authority keys (*PK*_*i*_, *SK*_*i*_) where *SK*_*i*_≔{***X***, *τ*, *σ*}.
• **Key generation** (*PP*, *i*, *SK*_*i*_, {*PK*}, *GID*, ***v***) → *sk*_*i*,*GID*,***v***_: It takes as input the global public parameters *PP*, the authority index *i*, its secret key *SK*_*i*_, all the public keys {*PK*_*i*_}_*i*∈[*n*]_, and *DU*’s global identifier *GID* and the attribute vector ***v***, and outputs the secret keys *sk*_*i*,*GID*,***v***_≔{*K*_*j*_}_*j*∈𝕊_*i*__.
• **Encryption** (*PP*, {*PK*}, ***x***, *F*) → *CT*_*F*_: It takes as inputs the global parameters *PP*, the public keys of all the authorities {*PK*_*i*_}_*i*∈[*n*]_, the ciphertext policy vector ***x*** and a file *F*, and outputs a ciphertext *CT*_*F*_.
• **Decryption** ({*sk*_*i*,*GID*,***v***_}_*i*∈*n*_, *CT*_*F*_) → *F*: It takes as inputs the collection of secret keys {*sk*_*i*,*GID*,***v***_} from *AA*_*i*_ and the ciphertext *CT*_*F*_, and outputs the message *F* if the access policy has been satisfied.

### Blockchain technology

Our proposed system integrates blockchain technology with MA-ABE and vector commitment mechanisms to advance decentralization. Blockchain was initially conceptualized by Nakamoto (2008) in 2009. It eliminates the need for a trusted third party (TTP) to oversee data management, favoring a distributed ledger maintained by consensus nodes. This ledger keeps track of a chronologically ordered list of transactions, which are validated *via* a consensus algorithm, such as proof of work (POW), before being permanently added to the ledger. In the Bitcoin system, miners solve complex cryptographic puzzles—a process known as PoW—to add blocks to the blockchain by packaging new transactions.

Ethereum (Wood, 2014), an evolution from the foundational Bitcoin, introduced a new platform for decentralized application with two distinct account types: external owned accounts (EOA) for the standard transaction and contract accounts for deploying self-executing and self-verifying protocols known as smart contracts. Each EOA is associated with a 20-byte hexadecimal address derived from the user’s public key (*BPK*), and transactions are authorized using the corresponding private key (*BSK*).

To address the storage scalability challenges of blockchains like Bitcoin and Ethereum (Nakamoto, 2008; Wood, 2014), the interplanetary file system (IPFS) (Benet, 2014) was developed. It is a peer-to-peer distributed file system offering content-addressed high-throughput storage, akin to a decentralized cloud service. When data is uploaded to IPFS, a unique hash of the file is generated, enabling users to access their data similarly to how URLs work on the traditional web.

## System Overview

### System architecture

The system comprises the following logical entities:

**Data owner** (*DO*): *DO* is an entity (individual or organization) that owns a certain file *F*. For secure storage and sharing, *DO* encrypts *F* using the AES key *AK* and uploads the encrypted file *CT*_*F*_ to the IPFS network, records the returned file location *loc*, and embeds *AK* and *loc* into the metadata *M* which is subsequently encrypted using the ABE system and published *CT*_*M*_ in the Ethereum network.

**Data user** (*DU*): *DU* is a data client for *DO*. It asks the attribute authority *AA* for permission to get the necessary attribute secret keys {*sk*}, which are then used to decrypt the associated *CT*_*M*_ stored on the Ethereum network. After getting the key *AK* and the location *loc* from *M*, *DU* can download the encrypted file *CT*_*F*_ from the IPFS network and recover the original file *F*.

**Attribute authority** (*AA*): *AA* is an entity (individual or organization) that contributes to the generation of the public parameters of the ABE system *PP*_*ABE*_ and the vector commitment scheme *PP*_*VC*_, publishes the public key *PK* for the *DO* to encrypt metadata *M*, owns a set of attributes and issues secret key *sk* for the owned attributes upon the request of the *DU*.

**Trusted attributed authority** (*AA*_trust_): *AA*_trust_ is a trusted attribute authority that mainly generates a secret key *sk* for *DU* and deploys system contracts for setup and registration. *AA*_trust_, unlike normal *AA*, owns no attributes but is in charge of a specific position in the attribute vector ***v***. It is important to note that, similar to our proposal, the scheme in Michalevsky & Joye (2018) also includes a *AA*_trust_. While this introduces a central element into the system, it is necessary for certain administrative tasks and security assurances.

**Service user** (*SU*): In the system, *SU* is a general entity comprising *DO*, *DU* and *AA*.

**Participant** (*P*): *P* is a special entity that represents each *AA* during the process of **Trusted Setup**. The index *i* of *P*_*i*_ denotes the chronological order of each piece of public parameter generated and shared by *AA*. **Blockchain**: Each user (*DO, DU, AA* and *AA*_trust_) possesses a pair of keys (*BPK*, *BSK*) and a corresponding wallet address *addr* on the blockchain. Our system employs two blockchains: IPFS for data storage and Ethereum for data governance.

and contracts:

**Trust setup contract** (*SC*_*sys*_): The contract *SC*_*sys*_ is deployed to the Ethereum network by the *AA*_trust_ and can only be invoked by an authorized *AA* within the time window specified. It is responsible for generating the global public parameters *PP*_*ABE*_.

**Authority setup contract** (*SC*_*auth*_): Contract *SC*_*auth*_ is deployed to the Ethereum network by the *AA*_trust_. It can only be invoked by the authorized *AA* within the specified time window. It is used to generate the global public parameters *PP*_*VC*_ and to keep track of the valid information about *AA*’s address *addr*, public key *PK*, and supported attributes $\mathcal{S}$.

**User registration contract** (*SC*_*reg*_): Contract *SC*_*reg*_ is deployed to the Ethereum network by the *AA*_trust_ and can be invoked by all the potential *DU*s. To register the *addr* in the system, *DU* needs to make sufficient payment to the *SC*_*reg*_ and then get back the *GID* which can later be used to request secret key *sk* from *AA*.

**Utility contract** (*SC*_*util*_): Contract *SC*_*util*_ is deployed to the Ethereum network by the *AA*_trust_ and can only be invoked by other contracts deployed by *AA*_trust_. It is mainly used to verify group elements published by *AA*.

**Log contract** (*SC*_*log*_): Contract *SC*_*log*_ is deployed to the Ethereum network by the *AA*_trust_. When it receives a new transaction from *DO*, it records the encrypted data *CT*_*M*_ of metadata *M* and triggers the event to the subscribers.

#### Architecture

**Figure 1:**
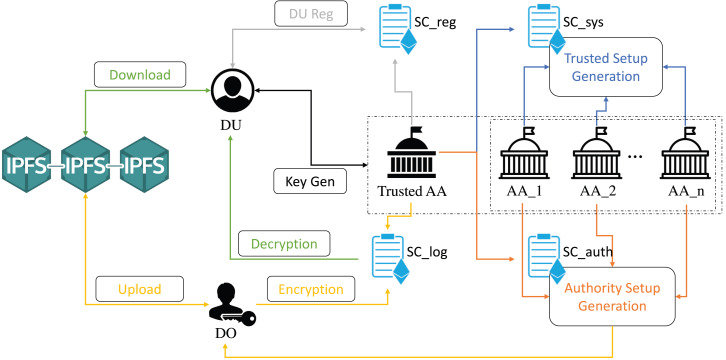
The system consists of six processes, each of which is represented by a different color: Blue for the process **Trusted Setup**, orange for the process **Authority Setup**, gray for the process **Data User Registration**, black for the process **Key Generation**, yellow for the process **Encryption and Upload**, and green for the process **Download and Decryption**. Single or double-arrow connectors indicate the interactions between service users and two blockchain networks, Ethereum and IPFS. Note that these four contracts deployed on Ethereum are used for data governance, while IPFS is used for data storage. For a detailed description of the system flow between smart contracts, IPFS, and various entities, please check “System Overview” for the Interaction Overview and “System Design” for the System Design.

The system architecture is shown in Fig. 1.

### Interactions overview

In this section, we describe the overview of our proposed system to show how smart contracts, IPFS, vector commitment, and MA-ABE with policy-hiding are composed together to build a secure, privacy-preserving, and blockchain-enabled data governance system. When it ought to be clear from the context, we omit most indices, like *i* and *j* of elements, and superscript in ${\mathbi{rp}}_{s}^{2}$ for readability.

1. **Trusted setup**
  1 First, a community of normal attribute authorities (*AA*s) with size *n* − 1 and a special trusted attribute authority (*AA*_trust_) must be determined. *AA*_trust_ selects the security parameter *λ* and two generators *g*_1_, *g*_2_ for the bilinear mapping, and defines following algorithms: COMMIT, NIZK, VerifyNIZK and powerMulti.
  2 *AA*_trust_ deploys one system contract *SC*_*sys*_ and one utility contract *SC*_*util*_.
  3 Each *AA* randomly samples a set of secret elements *e*: two matrixes $\mathbi{A}\in {\mathbb{Z}}_{p}^{(k+1)\times k}$ and $\mathbi{U}\in {\mathbb{Z}}_{p}^{(k+1)\times (k+1)}$, two scalars *α*_***A***_ and *α*_***U***_ in ${\mathbb{Z}}_{p}^{\ast }$, and two scaled matriex *α*_***A***_⋅***A*** and *α*_***U***_⋅***U***, and publishes a corresponding set of *s-pair* {***rp***_***A***_, ***rp***_***U***_, ***rp***_*α*_***A***__, ***rp***_*α*_***U***__, ***rp***_*α*⋅***A***_, ***rp***_*α*⋅***U***_} as defined in Definitions 2 and 3 to the contract *SC*_*sys*_.
  4 After that, *AA* computes and publishes the commitments *h*≔*COMMIT*({*h*_*s*_}||) to *SC*_*sys*_, where *h*_*s*_≔*COMMIT*(***rp***_*s*_), *s* ∈ *e*.
  5 Every *AA* then needs to prove the knowledge of each element *s* ∈ *e* by outputting the proofs {*π*_*s*_} using algorithm NIZK ( Algorithm 2 ) as the argument of function Prove of contract *SC*_*sys*_, which verifies them using algorithm VerifyNIZK ( Algorithm 3 ).
  6 In the **Round 1**, we define one attribute authority *AA* as participant *P*_1_ who firstly publishes group elements in 𝔾_1_: $({V}_{1}\coloneq {g}_{1}^{{\mathbi{A}}_{1}},{\theta }_{{V}_{1}}\coloneq {g}_{1}^{{\alpha }_{{\mathbi{A}}_{1}}},{V}_{1}^{{^{\prime}}}\coloneq {g}_{1}^{{\alpha }_{{\mathbi{A}}_{1}}\cdot {\mathbi{A}}_{1}})$, based on the previously verified set of elements *e*.
  7 Participant *P*_*i*=2,…,*n*_ computes $({V}_{i},{\theta }_{{V}_{i}},{V}_{i}^{{^{\prime}}})$ based on previous $({V}_{i-1},{\theta }_{{V}_{i-1}},{V}_{i-1}^{{^{\prime}}})$ using algorithm powerMult (Alg. 6) and publishes these as the arguments of the function Compute of contract *SC*_*sys*_ to check validity.
  8 We define the last valid *V* received by contract *SC*_*sys*_ as one piece of the public parameter ${g}_{1}^{\mathbi{A}}$.
  9 In the **Round 2**, the first *AA*, also known as participant *P*_1_, publishes group elements in 𝔾_1_: $({W}_{1}\coloneq (({g}_{1}^{\mathbi{A}})^{\top })^{{\mathbi{U}}_{1}},{\theta }_{{W}_{1}}\coloneq {g}_{1}^{{\alpha }_{{\mathbi{U}}_{1}}},{W}_{1}{^{\prime}}\coloneq (({g}_{1}^{\mathbi{A}})^{\top })^{{\alpha }_{{\mathbi{U}}_{1}}{\mathbi{U}}_{1}})$, also based on the previously verified set of elements *e*.
  10 Participant *P*_*i*_, where *i* = 2, …, *n* computes its $({W}_{i},{\theta }_{{W}_{i}}^{{^{\prime}}},{W}_{i}^{{^{\prime}}})$ based on previous $({W}_{i-1},{\theta }_{{W}_{i-1}}^{{^{\prime}}},{W}_{i-1}^{{^{\prime}}})$ using algorithm powerMult and publishes these as the arguments of the function Generate.
  11 We also define the last valid *W* received by contract *SC*_*sys*_ as last piece of the public parameter ${g}_{1}^{{\mathbi{U}}^{\top }\mathbi{A}}$. Therefore, we have $P{P}_{ABE}\coloneq \{ {g}_{1},{g}_{2},{g}_{1}^{\mathbi{A}},{g}_{1}^{{\mathbi{U}}^{\top }\mathbi{A}}\} $.
2. **Authority setup**
  1 *AA*_trust_ deploys contract *SC*_*auth*_ for authority setup.
  2 Each *AA* randomly samples another set of secret element *e*′: a matrix $\mathbi{X}\in {\mathbb{Z}}_{p}^{(k+1)\times (k+1)}$, a vector $\tau \in {\mathbb{Z}}_{p}^{k+1}$, two numbers $\sigma ,z\in {\mathbb{Z}}_{p}^{\ast }$, a scalar *α*_*z*_ and a scaled number *α*_*z*_⋅*z*. Using that, *AA* takes *SK*≔{***X***, *τ*, *σ*} as secret keys, and publishes a corresponding set of *s-pair* {***rp***_***X***_, ***rp***_*τ*_, ***rp***_*σ*_, ***rp***_*z*_, ***rp***_*α*_*z*_⋅*z*_} to the contract *SC*_*auth*_.
  3 After that, *AA* computes and publishes the commitment *h*′≔COMMIT({*h*_*s*_}||) to *SC*_*auth*_, where *h*_*s*_≔COMMIT(***rp***_*s*_), *s* ∈ *e*′.
  4 Every *AA* then needs to prove the knowledge of elements *s* ∈ *e*′ by generating the proofs {*π*_*s*_} using algorithm **NIZK** as the argument of function Prove of contract *SC*_*auth*_ for validity check.
  5 We define each *AA* with index *i* ∈ [*n* − 1] based on the receiving order of the complete set of valid {*π*_*s*_}_*s*∈*e*′_ and set attribute authority *AA*_trust_ with index *n*.
  6 Therefore, we have the verified sets of elements $P{K}_{i}\coloneq \{ {g}_{1}^{{\mathbi{X}}_{i}^{\top }\cdot \mathbi{A}},\hat {e}({g}_{1}^{{\tau }_{i}^{\top }\mathbi{A}},{g}_{2}),{g}_{2}^{\sigma }\} $ and $\{ {o}_{i}\coloneq {g}_{1}^{{z}_{i}},{g}_{1}^{{\alpha }_{{z}_{i}}},{g}_{1}^{{\alpha }_{{z}_{i}}\cdot {z}_{i}}\} $ for each *AA*_*i*_.
  7 In the last stage, for each *i*, *j* ∈ [*n*], *j* ≠ *i*, *AA*_*i*_ needs to compute a set of group elements in ${\mathbb{G}}_{1}:({O}_{i}\coloneq \{ ({o}_{j})^{{z}_{i}}\} ,{\theta }_{{O}_{i}}\coloneq \{ ({g}_{1}^{{\alpha }_{{z}_{j}}})^{{\alpha }_{{z}_{i}}}\} ,{O}_{i}^{{^{\prime}}}\coloneq \{ ({g}_{1}^{{\alpha }_{{z}_{j}}\cdot {z}_{j}})^{{\alpha }_{{z}_{i}}\cdot {z}_{i}}\} $. Then *AA*_*i*_ publishes these elements, with the number of supported attributes *l*_*i*_ as the argument of the function Setup.
  8 The contract *SC*_*auth*_ checks the validity of these elements published by *AA*_*i*_, *i* ∈ [*n*] and then registers its address *addr*_*i*_ with the elements (*l*_*i*_, *PK*_*i*_).
  9 In the end, we have *PP*_*VC*_≔{*g*_1_, *g*_2_, {*o*_*i*_}_*i*_, {*o*_*i*,*j*_}} where *i*, *j* ∈ [*n*], *i* ≠ *j* for vector commitment scheme.
3. **Data user registration**
  1 *AA*_trust_ deploys contract *SC*_*reg*_ for service registration.
  2 Data User (*DU*) makes a direct registration payment to the contract *SC*_*reg*_ to get the global identifier *GID* which is the hash value of DU’s address *addr*.
  3 Afterwards, *DU* can setup a secure channel with each *AA*_*i*_, *i* ∈ [*n*] that possesses the needed attributes and can verify *DU*’s identity.
  4 *AA*_*i*_ verifies *DU*’s identity and sends back the set of acknowledged attributes ${\mathcal{R}}_{i,GID}$ through the secure channel.
  5 Upon receiving all the ${\mathcal{R}}_{i,GID}$ from *AA*_*i*_, *DU* defines a set of ‘N/A’ attributes ${\mathcal{R}}_{j,GID}$ for those *AA*_*j*_, *i* ≠ *j* can not issue the attribute set and finally gets a complete set of attributes ${\mathcal{R}}_{GID}$ by combing ${\mathcal{R}}_{i,GID}$ and ${\mathcal{R}}_{j,GID}$ together.
4. **Key Gen**
  1 *DU* generates an attribute vector ***v***_*GID*_ from set of attributes ${\mathcal{R}}_{GID}$, creates a vector commitment ***C*** for ***v***_*GID*_ and sends it with opening proof *op*_*i*_ to each *AA*_*i*_, *i* ∈ [*n*] through separate secure channels.
  2 *AA*_*i*_, *i* ∈ [*n*] firstly checks the validity of its responsible part in the commitment ***C*** using *op*_*i*_, then issues *DU*’s requested attribute secret key *sk*_*i*,*GID*,***C***_, and finally sends it back to *DU* through the channel.
  3 Upon receiving responses from each *AA*_*i*_, *i* ∈ [*n*], *DU* gets a complete set of secret keys {*sk*_*i*,*GID*,***C***_}_*i*∈[*n*]_.
5. **Encryption and upload**
  1 *AA*_trust_ deploys last system contract *SC*_*log*_ to record encrypted related information of file *F*
  2 Data Owner (*DO*) randomly samples an AES key *AK*, encrypts *F* to obtain the ciphertext *CT*_*F*_, and uploads it to the IPFS network.
  3 After successfully receiving the *CT*_*F*_ from *DO*, IPFS network returns a special hash value *loc* as a file location on the IPFS network.
  4 Then, *DO* constructs a metadata *M*≔(*K*, *loc*), specifies a policy vector ***x*** based on selected attributes from each *AA*_*i*_, uses published {*PK*_*i*_} to encrypt the metadata *M* and publishes this encrypted information *CT*_*M*_ to contract *SC*_*log*_.
6. **Download and decryption**
  1 *DU* reads every new coming *CT*_*M*_ from the contract *SC*_*log*_ and checks if its owned secret keys {*sk*_*i*,*GID*,**C**_}, where *i* ∈ [*n*], satisfies the access policy ***x*** to recover the metadata *M*.
  2 *DU* retrieves the file location *loc* and AES key *AK* from the metadata *M* and requests the ciphertext *CT*_*F*_ from the IPFS network with the file location *loc*.
  3 *DU* uses the AES key *AK* to recover the original file *F*.

## System Design

In this section, we provide more details on the processes of **Trusted Setup**, **Authority Setup**, **Data User Registration**, **Key Generation**, **Encryption and Upload**, and **Download and Decryption**.

The code for the MA-ABE scheme with Non-interactive Zero-Knowledge Proof in **Auth Setup** is available on GitHub: https://github.com/Guy1m0/Attack-on-IPPE, but it does not include the code for any contracts, since the actual implementation may vary based on the version of the Solidity compiler used, which might affect the performance and gas cost of each contract.

Before the start of **Trusted Setup**, the trusted authority (*AA*_trust_) deploys the utility contract *SC*_*util*_.

### Trusted Setup

The process of **Trusted Setup** consists of four stages: *Initiate*, *Commit and Reveal*, *Verify*, and *Generate*, and finally outputs the global public parameter *PP*_*ABE*_ for the ABE system.

In the initial three stages, each attribute authority (*AA*) sends its transactions independently to the contract *SC*_*sys*_. In contrast, during the final *Generate* stage, each participant *P* (where we use the placeholder notation *P* in *Commit and Reveal* to represent each *AA*) must send transactions to *SC*_*sys*_ in a sequential manner. This sequentiality is necessary because each incoming transaction is generated based on the preceding *P*’s transaction received by contract *SC*_*sys*_.

#### Initiate

At the start, a fixed-numbered community of size *n* will be determined, which will include all of the normal attribute authorities *AA* and one special trusted authority *AA*_trust_. *AA*_trust_ represents this community to set the global security parameter to be *λ* and the generators of the 𝔾_1_, 𝔾_2_ with prime order *p* to be *g*_1_, *g*_2_ respectively. Therefore, the bilinear map can be $\hat {e}:{\mathbb{G}}_{1}\setminus \{ 0\} \times {\mathbb{G}}_{2}\setminus \{ 0\} \rightarrow {\mathbb{G}}_{T}$.

*AA*_trust_ also deploys two distinct system contracts: contract *SC*_*sys*_ for trusted setup and contract *SC*_*util*_ for resolving the problem of allowing complex cryptographic computations to be used in the system. *AA*_trust_ also specifies the deadlines (*ddl*_1_, *ddl*_2_, *ddl*_3_) for the **Trusted Setup** process and sets an authorized list *AAlist* to restrict *SC*_*sys*_ access. For a simple system description, we assume that each attribute authority *AA* submits the required transactions within the deadlines.

To realize the generation of *PP*_*ABE*_, this process highly depends on the interaction between each attribute authority *AA* and contract *SC*_*sys*_, which has five main functions, Commit, Reveal, Prove, Compute and Generate with the help from contract *SC*_*utils*_. Generally, these functions can only be invoked by a blockchain address owned by *AA*, which is included in the authorized list *AAlist*, and executed before the pre-defined deadline *ddl*_1,2,3_.

#### Commit and reveal

Every *AA* randomly picks a set of elements *e*: a matrix $\mathbi{A}\leftarrow _{}^{\text{$}}\{ \text{Diagonal matrices in}~{\mathbb{Z}}_{p}^{k\times k}\} \cup \left( \begin{array}{@{}c@{}} \displaystyle \mathbf{1} \end{array} \right) $, a matrix $\mathbi{U}\leftarrow _{}^{\text{$}}{\mathbb{Z}}_{p}^{(k+1)\times (k+1)}$^1^, 1The value of *k* in this context does not derive from the security parameter *λ*. Rather, it is a reference to Assumption 3.their corresponding scalar values, *α*_***A***_ and *α*_***U***_, and scaled matrixes *α*_***A***_⋅***A***, *α*_***U***_⋅***U***. Then, *AA* has

(3) \begin{eqnarray*}e=\{ \mathbi{A},\mathbi{U},{\alpha }_{\mathbi{A}},{\alpha }_{\mathbi{U}},{\alpha }_{\mathbi{A}}\cdot \mathbi{A},{\alpha }_{\mathbi{U}}\cdot \mathbi{U}\} \end{eqnarray*}

and then generates a set of *s-pair*.

For such element *s* ∈ *e*, we refer to the *s-pair* in 𝔾_1_ by ***rp***_*s*_ and in 𝔾_2_ by ${\mathbi{rp}}_{s}^{2}$ as Definition 2. These *s-pair* in both 𝔾_1_ and 𝔾_2_ are listed as follows:

• For matrix ***A***: $({\mathbi{rp}}_{\mathbi{A}},{\mathbi{rp}}_{\mathbi{A}}^{2})=(g,{g}^{\mathbi{A}})$
• For matrix ***U***: $({\mathbi{rp}}_{\mathbi{U}},{\mathbi{rp}}_{\mathbi{U}}^{2})=({g}^{{\mathbi{A}}^{\top }},{g}^{{\mathbi{A}}^{\top }\mathbi{U}})$
• For scalar *α*_***A***_: $({\mathbi{rp}}_{{\alpha }_{\mathbi{A}}},{\mathbi{rp}}_{{\alpha }_{\mathbi{A}}}^{2})=(g,{g}^{{\alpha }_{\mathbi{A}}})$
• For scalar *α*_***U***_: $({\mathbi{rp}}_{{\alpha }_{\mathbi{U}}},{\mathbi{rp}}_{{\alpha }_{\mathbi{U}}}^{2})=(g,{g}^{{\alpha }_{\mathbi{U}}})$
• For scaled matrix *α*_***A***_***A***: $({\mathbi{rp}}_{{\alpha }_{\mathbi{A}}\mathbi{A}},{\mathbi{rp}}_{{\alpha }_{\mathbi{A}}\mathbi{A}}^{2})=(g,{g}^{{\alpha }_{\mathbi{A}}\cdot \mathbi{A}})$
• For scaled matrix *α*_***U***_***U***: $({\mathbi{rp}}_{{\alpha }_{\mathbi{U}}\mathbi{U}},{\mathbi{rp}}_{{\alpha }_{\mathbi{U}}\mathbi{U}}^{2})=({g}^{{\mathbi{A}}^{\top }},{g}^{{\alpha }_{\mathbi{U}}\cdot {\mathbi{A}}^{\top }\mathbi{U}})$

Other than these *s-pair* listed above, *AA* also commits each element *s* ∈ *e*. For each *s* ∈ *e*:

\begin{eqnarray*}{h}_{s}\coloneq \text{COMMIT}({\mathbi{rp}}_{s}{|}{|}{\mathbi{rp}}_{s}^{2}) \end{eqnarray*}

Subsequently, the overall commitment is:

(4) \begin{eqnarray*}h\coloneq \text{COMMIT}({h}_{\mathbi{A}}{|}{|}{h}_{\mathbi{U}}{|}{|}{h}_{{\alpha }_{\mathbi{A}}}{|}{|}{h}_{{\alpha }_{\mathbi{U}}}{|}{|}{h}_{{\alpha }_{\mathbi{A}}\mathbi{A}}{|}{|}{h}_{{\alpha }_{\mathbi{U}}\mathbi{U}})\end{eqnarray*}

After that, *AA* publishes the commitment *h* to the contract *SC*_*sys*_ through blockchain transaction by calling function Commit, which works similarly as a hash function.

The state variable *h*_*collector* of *SC*_*sys*_ will store the value *h* with the key as *msg*.*sender*, also known as *AA*’s blockchain address. Apart from *h*_*collector*, we define few state variables used in contract *SC*_*sys*_ as follows:

1. *h*_*collector* (State Variable): A mapping collection from the blockchain address belonged to one attribute authority to its commitment *h*
2. *unverified*_*elements* (State Variable): A mapping collection from the blockchain address belonged to one attribute authority to its unverified list of *s-pair* s $L=\{ ({\mathbi{rp}}_{s},{\mathbi{rp}}_{s}^{2}){|}s\in e\} $ in both 𝔾_1_ and 𝔾_2_
3. *verified*_*elements* (State Variable): A mapping collection from the blockchain address belonged to one attribute authority to its verified list of *s-pair* s $L=\{ ({\mathbi{rp}}_{s},{\mathbi{rp}}_{s}^{2}){|}s\in e\} $ in both 𝔾_1_ and 𝔾_2_

After *h* has been received by contract *SC*_*sys*_, the sender needs to reveal committed element *s* ∈ *e* by passing a list of *s-pair* in both 𝔾_1_ and 𝔾_2_

(5) \begin{eqnarray*}{L}_{rp}=\{ ({\mathbi{rp}}_{s},{\mathbi{rp}}_{s}^{2}){|}s\in e\} \end{eqnarray*}

as argument of the function Reveal (Algorithm 4) before deadline *ddl*_1_, which checks the existence of the *h* published by *msg*.*sender*, and verifies that indeed *h* = *COMMIT*({*h*_*s*_}||)^2^ 2For brevity, we will use this shorthand notation to represent the above concatenation where *s* takes on all value in the set *e* = {***A***, ***U***, *α*_***A***_, *α*_***U***_, *α*_***A***_***A***, *α*_***U***_***U***}.as follows:

 
________________________________________________________________________________________________________________ 
Algorithm 4 Contract SCsys: Part 1_______________________________________________________________________________ 
  1:  function REVEAL(Lrp) 
  2:       for all (a,b) ∈ Lrp do 
 3:            h ← h|| HASH(a) || HASH(b) 
  4:       end for 
 5:       if msg.sender / ∈ unverified_elements then                             ⊳ Resubmitting check 
  6:            unverified_elements[msg.sender] ← Lrp 
  7:       end if 
 8:  end function 
 9: 
10:  function PROVE(Lπ) 
11:       Lrp ← unverified_elements[msg.sender] 
12:       for i ← 0,5 do 
13:            (rp,rp2) ← Lrp[i] 
14:            pi ← Lπ[i] 
15:            if not SAMERATIO(rp,rp2) then 
16:                  throw 
17:            end if 
18:            htmp ← h_collector[msg.sender]||HASH(rps) 
19:            if not CHECKPOK(rps,πs,htmp) then 
20:                  throw 
21:            end if 
22:       end for 
23:       verified_elements[msg.sender] ← Lrp 
24:       unverified_elements[msg.sender] = [] 
25:  end function___________________________________________________________________________________________    

Finally, each pair $({\mathbi{rp}}_{s},{\mathbi{rp}}_{s}^{2}),s\in e$ will be stored in another state variable *unverified*_*elements* with the key as *msg*.*sender*.

#### Verify

After deadline *ddl*_1_ set by *AA*_trust_ in the first stage *Initiate*, the system enters into the stage *Verify*. In this stage, we need to check that each attribute authority *AA* possesses the knowledge of the exponent *s* used in the list of *s-pair L*.

Every *AA* generates the proof *π*_*s*_≔*NIZK*(***rp***_*s*_, *h*||*h*_*s*_) using Algorithm 2 for each *s* ∈ *e*, and broadcasts these proofs as a list

(6) \begin{eqnarray*}{L}_{\pi }=\{ {\pi }_{\mathbi{A}},{\pi }_{\mathbi{U}},{\pi }_{{\alpha }_{\mathbi{A}}},{\pi }_{{\alpha }_{\mathbi{U}}},{\pi }_{{\alpha }_{\mathbi{A}}\cdot \mathbi{A}},{\pi }_{{\alpha }_{\mathbi{U}}\cdot \mathbi{U}}\} \end{eqnarray*}

through a blockchain transaction to get them verified. The function Prove ( Algorithm 4 ) from contract *SC*_*sys*_ takes input *L*_*π*_ and processes this verification work.

As shown above, it firstly calls function SameRatio, similar to Algorithm 1 , of contract *SC*_*util*_ to examine the authenticity of the published ***rp***_*s*_ and ${\mathbi{rp}}_{s}^{2}$.

Afterwards, it computes *h*_*tmp*_≔*h*||*COMMIT*(***rp***_*s*_), and takes *h*_*tmp*_ with verified ***rp***_*s*_ and provided *π*_*s*_ as input to the function CheckPoK of contract *SC*_*util*_, which works similarly as Algorithm 3 and returns *true* if the given proof *π*_*s*_ is valid.

Finally, the function Prove ( Algorithm 4 ) will remove the list of *s-pair L*_*rp*_ from the state variable *unverified*_*elements* and store it in the state variable *verified*_*elements*. This indicates that the *AA* possesses knowledge of the exponents for the set of *s-pair*.

#### Compute and generate

In this stage, the system will generate the public parameters for the attribute-based encryption in two rounds by interacting with two functions Compute and Generate of contract *SC*_*sys*_ (Algorithm 5). Some state variables used are defined as follows:

1. *V*_*curr* (State Variable): The most recent published value *V*
2. *V*_*curr*′ (State Variable): The most recent published value *V*′
3. *θ*_*curr* (State Variable): The most recent published scalar *θ*
4. *W*_*curr* (State Variable): The most recent published value *W*
5. *W*_*curr*′ (State Variable): The most recent published value *W*′
6. *θ*_*curr*_ (State Variable): The most recent published scalar *θ*

We also use below notation powerMulti (*A*, *B*) for the following Algorithm 6 :

 
________________________________________________________________________________________________________________ 
Algorithm 5 Contract SCsys: Part 2_______________________________________________________________________________ 
26:  function COMPUTE(V,θ,V ′) 
27:      if unverified_elements[msg.sender] ⁄= [] OR block.timestamp < ddl2 OR block.timestamp > ddl3 then 
28:           throw 
29:      end if 
30:      if not V _curr then                                                                               ⊳ Initialize V,θ,V ′ 
31:           V _curr ← V
32:           θ_curr ← θ 
33:           V _curr′ ← V ′ 
34:           return 
35:      end if 
36:      (rpA,rpαA,rpαAA) ← verified_elements[msg.sender] 
37:      acc1 ←SAMERATIO((V _curr,V ),rpA) 
38:      acc2 ←SAMERATIO((θ_curr,θ),rpαA) 
39:      acc3 ←SAMERATIO((V _curr′,V ′),rpAαA) 
40:      if acc1 == acc2 == acc3 == true  then                                                          ⊳ Check validity 
41:           V _curr ← V
42:           θ_curr ← θ 
43:           V _curr′ ← V ′ 
44:      else 
45:           throw 
46:      end if 
47:  end function 
48: 
49:  function GENERATE(W,θ,W′) 
50:      if unverified_elements[msg.sender] ⁄= [] OR block.timestamp < ddl2 OR block.timestamp > ddl3 then 
51:           throw 
52:      end if 
53:      if not W _curr then                                                                             ⊳ Initialize W,θ,W′ 
54:           W _curr ← W 
55:           θ_curr_ ← θ 
56:           W _curr′ ← W′ 
57:           return 
58:      end if 
59:      (rpU,rpαU ,rpαUU) ← verified_elements[msg.sender] 
60:      acc1 ←SAMERATIO((W _curr,W),rpU) 
61:      acc2 ←SAMERATIO((θ_curr_,θ),rpαU ) 
62:      acc3 ←SAMERATIO((W _curr′,W′),rpαUU) 
63:      if acc1 == acc2 == acc3 == true then                                                          ⊳ Check validity 
64:           W _curr ← W 
65:           θ_curr_ ← θ 
66:           W _curr′ ← W′ 
67:      else 
68:           throw 
69:      end if  
70:  end function______________________________________________________________________________________________________________    

 
____________________________________________________________________________________________________________________________________ 
Algorithm 6 Computing power matrix A by matrix B_______________________________________________ 
Require: group elements A and matrix s have same size l × k 
 1:  function POWERMULTI(A,s) 
  2:       for i ← 1,l do 
 3:            for j ← 1,k do 
 4:                  B[i,j] ← A[i,j]s[i,j] 
  5:            end for 
 6:       end for 
 7:       return B 
 8:  end function___________________________________________________________________________________________ 

**Round 1:** We define the first attribute authority *AA* as participant *P*_1_, who broadcasts $({V}_{1},{\theta }_{{V}_{1}},{V}_{1}^{{^{\prime}}})$ as argument of function Compute ( Algorithm 5 ) in contract *SC*_*sys*_. The elements $({V}_{1},{\theta }_{{V}_{1}},{V}_{1}^{{^{\prime}}})$ are constructed as follows: ${V}_{1}\coloneq {g}_{1}^{{\mathbi{A}}_{1}},{\theta }_{{V}_{1}}\coloneq {g}_{1}^{{\alpha }_{{\mathbi{A}}_{1}}},{V}_{1}^{{^{\prime}}}\coloneq {g}_{1}^{{\alpha }_{{\mathbi{A}}_{1}}\cdot {\mathbi{A}}_{1}}$. And the next participant *P*_*i*_,  *i* =2 , 3, …, *n*, generates corresponding elements $({V}_{i},{\theta }_{{V}_{i}},{V}_{i}^{{^{\prime}}})$ using Algorithm 6 , and also broadcasts them to the contract *SC*_*sys*_:

\begin{eqnarray*}{V}_{i}& \coloneq powerMulti({V}_{i-1},{\mathbi{A}}_{i}) \end{eqnarray*}

\begin{eqnarray*}{\theta }_{{V}_{i}}& \coloneq ({\theta }_{{V}_{i-1}})^{{\alpha }_{{\mathbi{A}}_{i}}} \end{eqnarray*}

\begin{eqnarray*}{V}_{i}^{{^{\prime}}}& \coloneq powerMulti({V}_{i-1}^{{^{\prime}}},{\alpha }_{{\mathbi{ A}}_{i}}{\mathbi{A}}_{i}) \end{eqnarray*}

Since receiving the elements $({V}_{1},{\theta }_{1},{V}_{1}^{{^{\prime}}})$ from first participant *P*_1_, function Compute ( Algorithm 5 ) of *SC*_*sys*_ checks the validity of each incoming elements $({V}_{i},{\theta }_{{V}_{i}},{V}_{i}^{{^{\prime}}})$ published by *P*_*i*_.

In the end, we define the last valid *V*_*i*_ as one piece of the public parameter:

\begin{eqnarray*}{g}_{1}^{\mathbi{A}}=powerMulti(powerMulti(\ldots (powerMulti(powerMulti({g}_{1}^{{\mathbi{A}}_{1}},{\mathbi{A}}_{2}),{\mathbi{A}}_{3})\ldots ,{\mathbi{A}}_{n}). \end{eqnarray*}

**Round 2:** In this round, we also define the first attribute authority *AA* as participant *P*_1_, who broadcasts $({W}_{1},{\theta }_{1}^{{^{\prime}}},{W}_{1}^{{^{\prime}}})$ as argument of the function Generate ( Algorithm 5 ) in contract *SC*_*sys*_. The elements $({W}_{1},{\theta }_{1}^{{^{\prime}}},{W}_{1}^{{^{\prime}}})$ are constructed as follows: ${W}_{1}\coloneq (({g}_{1}^{\mathbi{A}})^{\top })^{{\mathbi{U}}_{1}},{\theta }_{1}^{{^{\prime}}}\coloneq {g}_{1}^{{\alpha }_{{\mathbi{U}}_{1}}},{W}_{1}^{{^{\prime}}}\coloneq (({g}_{1}^{\mathbi{A}})^{\top })^{{\alpha }_{{\mathbi{U}}_{1}}{\mathbi{U}}_{1}}$. And the next participant *P*_*i*_ where *i* = 2, 3, …, *n*, generates corresponding elements $({W}_{i},{\theta }_{{W}_{i}},{W}_{i}^{{^{\prime}}})$ using Algorithm 6 , and also broadcasts them to the contract *SC*_*sys*_:

\begin{eqnarray*}{W}_{i}& \coloneq powerMulti({W}_{i-1},{\mathbi{U}}_{i}) \end{eqnarray*}

\begin{eqnarray*}{\theta }_{{W}_{i}}& \coloneq ({\theta }_{{W}_{i-1}})^{{\alpha }_{\mathbi{U},i}} \end{eqnarray*}

\begin{eqnarray*}{W}_{i}^{{^{\prime}}}& \coloneq powerMulti({W}_{i-1}^{{^{\prime}}},{\alpha }_{\mathbi{ U},i}{\mathbi{U}}_{i}) \end{eqnarray*}

Invoked by these transactions, contract *SC*_*sys*_ checks the validity of each received elements $({W}_{i},{\theta }_{{W}_{i}},{W}_{i}^{{^{\prime}}})$ and updates the value of *W*_*i*_.

As a result of this procedure, the final piece of the public parameter is defined as the last valid *W*_*i*_ received:

\begin{eqnarray*}{g}_{1}^{{\mathbi{U}}^{\top }\mathbi{A}}={W}_{n}=PowerMulti(PowerMulti(\ldots (\nonumber\\\displaystyle PowerMulti(PowerMulti((({g}_{1}^{\mathbi{A}})^{\top })^{{\mathbi{U}}_{1}},{\mathbi{U}}_{2}),\ldots ,{\mathbi{U}}_{n}))))^{\top } \end{eqnarray*}

At the end of this stage, each Service User (*SU*) can easily get the global public parameters *PP*_*ABE*_ of the attribute-based encryption system based on the published values.

(7) \begin{eqnarray*}P{P}_{ABE}\coloneq \{ {g}_{1},{g}_{2},{g}_{1}^{\mathbi{A}},{g}_{1}^{{\mathbi{U}}^{\top }\mathbi{A}}\} .\end{eqnarray*}

### Authority setup

This step consists of 3 stages: *Commit and Reveal*, *Verify*, and *Generate*, and finally outputs another global public parameter *PP*_*VC*_ and public key *PK* for each attribute authority *AA*.

#### Initiate

The contract *SC*_*auth*_ deployed by trusted authority *AA*_trust_ has four main functions, Commit, Reveal, Prove and Generate, which also interacts with utility function *SC*_*util*_. Its accessibility is also limited by deadlines (*ddl*_1_′, *ddl*_2_′, *ddl*_3_′) and the authorized list *AAlist* set by *AA*_trust_.

#### Commit and reveal

Every *AA* firstly samples a set of elements *e*′: a matrix $\mathbi{X}\leftarrow _{}^{\text{$}}{\mathbb{Z}}_{p}^{(k+1)\times (k+1)}$, a vector $\tau \leftarrow _{}^{\text{$}}{\mathbb{Z}}_{p}^{k+1}$, two secret elements $\sigma ,z\in {\mathbb{Z}}_{p}^{\ast }$, a scalar *α*_*z*_ and a scaled element *α*_*z*_⋅*z*.

Therefore, the *AA* defines a set *e*′ for vector commitment as:

\begin{eqnarray*}{e}^{{^{\prime}}}\coloneq \{ z,{\alpha }_{z},{\alpha }_{z}\cdot z\} \end{eqnarray*}

and also designates:

\begin{eqnarray*}SK\coloneq \{ \mathbi{X},\tau ,\sigma \} \end{eqnarray*}

as the secret key. Then, *AA* computes a set of *s-pair* for each element *s* in both *e*′ and *SK*. We refer to the *s-pair* in 𝔾_1_ by ***rp***_*s*_, and the *s-pair* in 𝔾_2_ by ${\mathbi{rp}}_{s}^{2}$ as Definition 2. These *s-pair* are defined as follows:

• For matrix ${\mathbi{X}}^{\top }:{\mathbi{rp}}_{\mathbi{X}}\coloneq ({g}_{1}^{\mathbi{A}},{g}_{1}^{{\mathbi{X}}^{\top }\cdot \mathbi{A}})$
• For vector ${\tau }^{\top }:{\mathbi{rp}}_{\tau }\coloneq ({g}_{1}^{\mathbi{A}},{g}_{1}^{{\tau }^{\top }\mathbi{A}})$
• For element $\sigma :{\mathbi{rp}}_{\sigma }^{2}\coloneq ({g}_{2},{g}_{2}^{\sigma })$
• For element $z:({\mathbi{rp}}_{z},{\mathbi{rp}}_{z}^{2})=(g,{g}^{z})$
• For scalar *α*_*z*_: $({\mathbi{rp}}_{{\alpha }_{z}},{\mathbi{rp}}_{{\alpha }_{z}}^{2})=(g,{g}^{{\alpha }_{z}})$
• For scaled element *α*_*z*_*z*: $({\mathbi{rp}}_{{\alpha }_{z}z},{\mathbi{rp}}_{{\alpha }_{z}z}^{2})=(g,{g}^{{\alpha }_{z}\cdot z})$

After that, *AA* computes *h*′ as follows:

(8) \begin{eqnarray*}\begin{array}{@{}l@{}} \displaystyle {h}_{\mathbi{X}}\coloneq \text{COMMIT}({\mathbi{rp}}_{\mathbi{X}}) {h}_{\tau }\coloneq \text{COMMIT}({\mathbi{rp}}_{\tau }) {h}_{\sigma }\coloneq \text{COMMIT}({\mathbi{rp}}_{\sigma }^{2})\\ \displaystyle {h}_{z}\coloneq \text{COMMIT}({\mathbi{rp}}_{z}{|}{|}{\mathbi{rp}}_{z}^{2}) {h}_{{\alpha }_{z}}\coloneq \text{COMMIT}({\mathbi{rp}}_{{\alpha }_{z}}{|}{|}{\mathbi{rp}}_{{\alpha }_{z}}^{2}) {h}_{{\alpha }_{z}\cdot z}\coloneq \text{COMMIT}({\mathbi{rp}}_{{\alpha }_{z}\cdot z}{|}{|}{\mathbi{rp}}_{{\alpha }_{z}\cdot z}^{2})\\ \displaystyle {h}^{{^{\prime}}}\coloneq \text{COMMIT}({h}_{\mathbi{X}}{|}{|}{h}_{\tau }{|}{|}{h}_{\sigma }{|}{|}{h}_{z}{|}{|}{h}_{{\alpha }_{z}}{|}{|}{h}_{{\alpha }_{z}\cdot z}) \end{array}\end{eqnarray*}

and broadcasts it to the contract *SC*_*auth*_ through blockchain transaction as the argument of the function Commit, which is exactly same as it of the contract *SC*_*sys*_. It will store the value *h*′ into state variable *h*_*collector* if the transaction is valid.

After *h*′ recorded by contract *SC*_*auth*_, *AA* needs to reveal each committed element by passing two lists of *s-pair*

(9) \begin{eqnarray*}{L}_{sk}& =\{ {\mathbi{rp}}_{\mathbi{X}},{\mathbi{rp}}_{\tau },{\mathbi{rp}}_{\sigma }^{2}\} \end{eqnarray*}

(10) \begin{eqnarray*}{L}_{{e}^{{^{\prime}}}}& =\{ ({\mathbi{rp}}_{s},{\mathbi{rp}}_{s}^{2}){|}s\in {e}^{{^{\prime}}}\} \end{eqnarray*}

as arguments of the function Reveal of the contract *SC*_*auth*_, which computes the hash result of *L*_*sk*_ and *L*_*vc*_ using function Hash of utility contract *SC*_*util*_, and compares the result with the value stored in state variable *h*_*collector*. Finally, the valid set of *s-pair* will be stored into state variables *unverified*_*elements* and *unverified*_*sk* of the contract *SC*_*auth*_ respectively.

#### Verify

The system enters into the stage *Verify* after $dd{l}_{1}^{{^{\prime}}}$ set by trusted authority *AA*_trust_.

First of all, attribute authority *AA* generates the proof *π*_*s*_≔*NIZK*(***rp***_*s*_, *h*||*h*_*s*_) for each *s* in both *e*′ and *SK*, and broadcast these proofs {*π*} as a list

(11) \begin{eqnarray*}{L}_{\pi }^{{^{\prime}}}=\{ {\pi }_{z},{\pi }_{{\alpha }_{z}},{\pi }_{{\alpha }_{z}z},{\pi }_{\mathbi{X}},{\pi }_{\tau },{\pi }_{\sigma }\} \end{eqnarray*}

through transaction before deadline $dd{l}_{2}^{{^{\prime}}}$. The function Prove of contract *SC*_*auth*_ takes input ${L}_{\pi }^{{^{\prime}}}$ as the argument and checks the validity of these proofs by using utility functions SameRatio and CheckPoK of contract *SC*_*utils*_. The Reveal-Prove algorithms in the contract *SC*_*auth*_ function similarly to those in contract *SC*_*sys*_. Both require all authorities to commit their secret keys and later prove that they possess the knowledge of these keys.

We assign each *AA* an index *i* ∈ [*n* − 1], based on the order in which *SC*_*auth*_ receives the complete set of valid published proofs {*π*}. The trusted authority, denoted as *AA*_trust_, is assigned the index *n*.

At the end of this stage, we have the verified elements $P{K}_{i}=({g}_{1}^{{\mathbi{X}}_{i}^{\top }\mathbi{A}},\hat {e}({g}_{1},{g}_{2})^{{\tau }_{i}^{\top }\mathbi{A}},{g}_{2}^{\sigma })$ and $\{ {o}_{i}\coloneq {g}_{1}^{{z}_{i}},{g}_{1}^{{\alpha }_{{z}_{i}}},{g}_{1}^{{\alpha }_{{z}_{i}}{z}_{i}}\} $ for each *AA*_*i*_.

#### Generate

In the last stage *Generate*, *AA*_*i*_ needs to generate a set of group elements (*O*, *θ*_*O*_, *O*′), selects a reasonable number of supported attributes *l*_*i*_, and broadcasts them to contract *SC*_*auth*_ before the deadline $dd{l}_{3}^{{^{\prime}}}$.

The elements (*O*, *θ*_*O*_, *O*′) provided by *AA*_*i*_ are constructed as follows:

\begin{eqnarray*}{O}_{i}& \coloneq {o}_{i,j}=\{ ({o}_{j})^{{z}_{i}}\} _{i\not = j,j\in [n]} \end{eqnarray*}

\begin{eqnarray*}{\theta }_{{O}_{i}}& \coloneq \{ ({g}_{1}^{{\alpha }_{{z}_{j}}})^{{\alpha }_{{z}_{i}}}\} _{i\not = j,j\in [n]} \end{eqnarray*}

\begin{eqnarray*}{O}_{i}^{{^{\prime}}}& \coloneq \{ ({g}_{1}^{{\alpha }_{{z}_{j}}{z}_{j}})^{{\alpha }_{{z}_{i}}{z}_{i}}\} _{i\not = j,j\in [n]} \end{eqnarray*}

 
________________________________________________________________________________________________________________ 
Algorithm 7 Contract SCauth__________________________________________________________________________________________ 
  1:  function GENERATE(O,θ,O′,l) 
  2:       if msg.sender / ∈ AAlist OR block.timestamp > ddl then              ⊳ Requirement check 
  3:            throw 
  4:       end if 
 5:       rpz,rpαz,rpαzz ← verified_elements[msg.sender] 
  6:       i ← index[msg.sender] 
  7:       for j ← 0,n − 1 do 
 8:            if i == j then 
 9:                  continue 
10:            end if 
11:            check1 ← SAMERATIO((rpz[1],O[j]),rpz) 
12:            check2 ← SAMERATIO((rpα[1],θ[j]),rpαz) 
13:            check3 ← SAMERATIO((rpαzz[1],O′[j]),rpαzz) 
14:            if check1 == check2 == check3 == true then 
15:                  verified_O[msg.sender] ← O 
16:            else 
17:                  throw 
18:            end if 
19:       end for 
20:       attribute_size[msg.sender] ← l 
21:  end function___________________________________________________________________________________________    

Invoked by this transaction, function Generate ( Algorithm 7 ) of *SC*_*auth*_ firstly checks the validity of elements $({O}_{i},{\theta }_{{O}_{i}},{O}_{i}^{{^{\prime}}})$ and the value of *l*_*i*_, and then records *AA*_*i*_’s blockchain address *addr* with the claimed attribute size *l*_*i*_. The function Generate and some state variables used are defined as follows:

1. *verified*_*O* (State Variable): A mapping collection from the blockchain address belonged to one attribute authority to its set of elements $(\{ {o}_{j}^{{z}_{i}}\} _{i\not = j,i,j\in [n]}$ in the public parameter of vector commitment *PP*_*VC*_
2. *attribute*_*size* (State Variable): A mapping collection from the blockchain address belonged to one attribute authority to its number of supported attribute

For example, if *AA*_*i*_ owns the set of attributes {*entry*, *mid*, *senior*, *agent*, *manager*}, the value of attribute size *l*_*i*_ is 5.

After the contract *SC*_*auth*_ receives all the pairs {*o*_*i*_, *o*_*i*,*j*_, *l*_*i*_} from *AA*_*i*_ ∈ {*AA*_1_, *AA*_2_, …, *AA*_*n*_}, every Service User (*SU*) can get the global parameter for the vector commitment system

(12) \begin{eqnarray*}P{P}_{VC}\coloneq \{ {g}_{1},{g}_{2},\{ {o}_{i}\} _{i\in [n]},\{ {o}_{i,j}\} _{i,j\in [n],i\not = j}\} \end{eqnarray*}

and generate a mapping table that maps each blockchain address of *AA*_*i*_ to its corresponding information as shown in Table 3. We use *l* to represent the size of supported attributes set $\mathcal{X}$ and *v* to represent the owned attributes for each *AA*.

**Table 3:** A example of address-*AA*-attribute vector mapping table.

Address	*addr*_1_		*addr*_2_	......	*addr*_*n*−1_		*addr*_*n*_
Attribute authority	*AA*_1_		*AA*_2_	......	*AA*_*n*−1_		*AA*_trust_
Attribute representation	*v*_1_	*v*_2_	*v*_3_	......	*v*_*l*−1_	*v*_*l*_	*v*_*l*+1_

### Data user registration

To get the global identifier *GID*, which will be used in the process of *Key Generation*, the data user (*DU*) needs to register the owned address *addr*_*DU*_ by calling the function user_register of the contract *SC*_*REG*_.

*DU* needs to send predefined amount of *GWEI* to the contract *SC*_*REG*_ as the registration fee payment. This amount defaults to 1,000,000 *GWEI*, which is approximately equivalent to 1.63 USD as of September 2023. In return, *DU* receives a value *GID*, which is the hash value of *DU*’s address *addr*_*DU*_, also known as the *msg*.*sender* of this transaction call.

Following that, *DU* establishes a secure channel or employs some off-chain methods with *AA*_*i*_ that have the required attributes and can verify *DU*’s identity. We assume that *DU* is an agent for one insurance company, and the company itself runs the consensus node of *AA*_*i*_ in this system. Therefore, *DU* may easily get verified by showing an ID badge to the person who manages the *AA*_*i*_. *DU* then requests that *AA*_*i*_ issues a set of attributes ${\mathcal{R}}_{i,GID}$ in regards to *DU*’s identity. The format of a set of attributes might look like this: (*entry*, *N*/*A*, *N*/*A*, *agent*, *N*/*A*) out of the full set of attributes (*entry*, *mid*, *senior*, *agent*, *manager*).

For those *AA*_*j*_, *j* ≠ *i* that do not contain the needed attributes or cannot verify *DU*’s identity, *DU* may just set ${\mathcal{R}}_{j,GID}$ to be (*N*/*A*, *N*/*A*, *N*/*A*) with *l*_*j*_ = 3.

Finally, *DU* receives ${\mathcal{R}}_{i,GID}$ from *AA*_*i*_, sets ${\mathcal{R}}_{j,GID}$ for *AA*_*j*_, *j* ≠ *i*, and combines them as the set of attributes ${\mathcal{R}}_{GID}$ which will be used in the process of Key Generation.

### Key generation

Without loss of generality, we suppose that there are a total set of attributes $\mathcal{X}$, indexed from 1 to *l* and a total set of attributes authorities $\mathcal{U}$ including *AA*_trust_, indexed from 1 to *n*. Assume that the attribute authority *AA*_*i*_ has a subset of attributes ${\mathcal{S}}_{i}$, then we have ${\mathcal{S}}_{i}&cap; {\mathcal{S}}_{j}={0}$ for *i* ≠ *j* and *i*, *j* ∈ |*n*| and ${\mathcal{S}}_{1}&cup; {\mathcal{S}}_{2}...&cup; {\mathcal{S}}_{n}=\mathcal{X}$.

To get the secret key *sk*_*i*,*GID*,***C***_ which is comprised of multiple key parts ${&lcub; {K}_{j,GID,\mathbi{C}}&rcub; }_{j&isin; {\mathcal{S}}_{i}}$ from *AA*_*i*_, Data User (*DU*) must initially generate the attribute vector ***v***_*GID*_. This is based on ${\mathcal{R}}_{GID}$ that was acquired during the **Data User Registration** process.

Given that the *DU*’s set of attributes ${\mathcal{R}}_{i,GID}&isin; \mathcal{X}$ and the mapping Table 3 generated from the process **Authority Setup**, the attribute vector ***v***_*GID*_ is set as follows:

1. Set the first *l* entries such that *v*_*k*_ = $ \left\{ {\scriptsize \begin{array}{@{}ll@{}} \displaystyle 1 &\displaystyle i\in {R}_{GID}\\ \displaystyle 0 &\displaystyle i\not \in {R}_{GID}\\ \displaystyle \end{array}} \right. $
2. Set the *l* + 1 entry to be 1. (*AA*_trust_ is responsible for this entry)

Then, *DU* randomly chooses ${r}_{1},{r}_{2},/dots,{r}_{n}\leftarrow _{}^{\text{$}}{\mathbb{Z}}_{p}^{\ast }$ for each *AA* and combines the arguments *r*_*i*_ with the attribute vector ***v***_*i*,*GID*_ (represented as a bit string) to produce a committed value *m*_*i*_≔COMMIT(*v*_*i*,1_||*v*_*i*,2_…*v*_*i*,*j*_||*r*_*i*_), where $i&isin; [n],j&isin; [{|}{\mathcal{S}}_{i}{|}]$.

**Table 4:** A example of *m* for vector commitment.

Attribute authority	*AA*_1_			*AA*_2_	
Attributes	entry	mid	senior	agent	manager
Vector element	*v*_1_	*v*_2_	*v*_3_	*v*_4_	*v*_5_
Element value	1	0	0	1	0
nonce	*r*_1_			*r*_2_	
*m*_*i*_	COMMIT (*v*_1_||*v*_2_||*v*_3_||*r*_1_)			COMMIT (*v*_4_||*v*_5_||*r*_2_)	

In Table 4, for instance, we have two attribute authorities *AA*_1_ and *AA*_2_ that possess attributes (entry, mid, senior) and (agent, manager) respectively. If there is a data user *DU* with a set of attributes $\mathcal{R}=$(entry, agent), *DU*’s attribute vector is ***v*** = (1, 0, 0, 1, 0). For *AA*_1_ and *AA*_2_, *DU*samples two random values ${r}_{1},{r}_{2}\leftarrow _{}^{\text{$}}{\mathbb{Z}}_{p}^{\ast }$ and then computes auxiliary information ***aux*** = (*m*_1_, *m*_2_) using COMMIT. In general, based on the public parameter for vector commitment *PP*_*VC*_ = {*g*_1_, *g*_2_, {*o*_*i*_}_*i*∈[*n*]_, {*o*_*i*,*j*_}_*i*,*j*∈[*n*],*i*≠*j*_}, generated in **Authority Setup**, *DU* can calculate the commitment ***C*** on the attribute vector ***v*** from its $\mathcal{R}$:

\begin{eqnarray*}\mathbi{C}\coloneq {o}_{1}^{{m}_{1}}{o}_{2}^{{m}_{2}}\ldots {o}_{n}^{{m}_{n}} \end{eqnarray*}

To request the secret key part ${K}_{j,GID,\mathbi{C}},j&isin; {\mathcal{S}}_{i}$, *DU* establishes another secure channel with *AA*_*i*_ and sends commitment *C* along with an opening *op*_*i*_ and nonce *r*_*i*_. Such opening *op*_*i*_ is calculated as follows:

\begin{eqnarray*}o{p}_{i}=\prod _{j=1,j\not = i}^{n}{o}_{i,j}^{{m}_{j}}=(\prod _{j=1,j\not = i}^{n}{o}_{j}^{{m}_{j}})^{{z}_{i}} \end{eqnarray*}

Based on the *DU*’s *GID*, *AA*_*i*_ firstly retrieves the information of the set of attributes ${\mathcal{R}}_{i,GID}$ which have been issued in the previous process **Data User Registration** and then verifies commitment ***C*** using the opening *op*_*i*_ and nonce *r*_*i*_ received

\begin{eqnarray*}\hat {e}(\mathbi{C}/{o}_{i}^{{m}_{i}^{{^{\prime}}}},{g}_{2}^{{z}_{i}})\stackrel{{?}}{=}\hat {e}(o{p}_{i},{g}_{2}) \end{eqnarray*}

where ${m}_{i}^{{^{\prime}}}$ is the value calculated by the ${\mathcal{R}}_{i,GID}$ issued to DU. If the above check passes, *AA*_*i*_ uses a pre-defined random oracle $\mathcal{H}:{\mathbb{G}}_{2}\times \{ 0,1\} ^{\lambda }\times {\mathbb{Z}}_{p}^{k+1}\rightarrow {\mathbb{Z}}_{p}^{k+1}$ to generate masking value ${\mu }_{\mathbi{i}}\in {\mathbb{Z}}_{p}^{\ast }$

\begin{eqnarray*}{\mu }_{\mathbi{i}}=\sum _{j=1}^{i-1}\mathcal{H}({y}_{j}^{{\sigma }_{i}},GID,\mathbi{C})-\sum _{j=i+1}^{n}\mathcal{H}({y}_{j}^{{\sigma }_{i}},GID,\mathbi{C}) \end{eqnarray*}

and hash functions *H*_1_(*GID*, ***C***), …, *H*_*k*+1_(*GID*, ***C***) to generate ${g}_{2}^{\mathbi{h}}$ where $\mathbi{h}\in {\mathbb{Z}}_{p}^{k+1}$

\begin{eqnarray*}\mathbi{h}:H(GID,\mathbi{C})=({H}_{1}(GID,\mathbi{C}),\ldots ,{H}_{k+1}(GID,\mathbi{C}))^{\top } \end{eqnarray*}

Finally, *AA*_*i*_ computes the secret keys

(13) \begin{eqnarray*}s{k}_{i,GID,\mathbi{C}}\coloneq \{ {K}_{j,GID,\mathbi{C}}\} _{j\in {\mathcal{S}}_{i}},\end{eqnarray*}

which consists of key parts

(14) \begin{eqnarray*}{K}_{j,GID,\mathbi{C}}\coloneq {g}_{2}^{{\tau }_{\mathbi{i}}}-{v}_{j}{\mathbi{X}}_{i}\mathbi{h}+{\mu }_{\mathbi{i}}\end{eqnarray*}

for each possessed attributes by *AA*_*i*_ and send *sk*_*i*,*GID*,***C***_ back to the *DU* through the secure channel.

To get the special secret key part *sk*_*n*,*GID*,***C***_ for the *l* + 1 entry, *DU* also needs to communicate with trusted authority *AA*_trust_ and provides the commitment ***C*** with the opening *op*_*n*_ and nonce *r*_*n*_ through the secure channel. As shown in the Table 3, *AA*_trust_ sets the ${m}_{n}^{{^{\prime}}}$ to be COMMIT ((*v*_*l*+1_||*r*_*n*_)) where *v*_*l*+1_ = 1 and then checks the following equation

\begin{eqnarray*}\hat {e}(\mathbi{C}/{o}_{n}^{{m}_{n}^{{^{\prime}}}},{g}_{2}^{{z}_{n}})\stackrel{{?}}{=}\hat {e}(o{p}_{n},{g}_{2}) \end{eqnarray*}

If it passes, *AA*_trust_ computes the secret key part similar as *sk*_*i*,*GID*,***C***_

(15) \begin{eqnarray*}s{k}_{n,GID,\mathbi{C}}\coloneq {K}_{l+1}={g}_{2}^{{\tau }_{n}}-{v}_{n}{\mathbi{X}}_{n}\mathbi{h}+{\mu }_{n}\end{eqnarray*}

and sends it back to *DU*.

Upon receiving all the responses from each *AA*_*i*_, *i* ∈ [*n*], *DU* will finally gets a complete set of secret keys {*sk*_*i*,*GID*,***C***_}_*i*∈[*n*]_.

### Encryption and upload

Given that ABE is significantly more expensive than symmetric key encryption (Wang et al., 2014), the files that the data owner (*DO*) wants to share are not directly encrypted with ABE. Instead, hybrid encryption of ABE and AES is used for efficiency.

Firstly, *DO* randomly samples an AES key *AK* from the key space and encrypts the file *F* as the ciphertext *CT*_*F*_.

Then, *DO* uploads the ciphertext *CT*_*F*_ to IPFS and records the file location *loc* returned by IPFS. The metadata message *M* can then be constructed as:

\begin{eqnarray*}M\coloneq (K,loc) \end{eqnarray*}

Using the policy vector ***x*** discussed above, *DO* samples a random vector $\mathbi{s}\in {\mathbb{Z}}_{p}^{k}$, generates a policy vector $\mathbi{x}\coloneq ({x}_{1},{x}_{2},\ldots ,{x}_{n})\in {\mathbb{Z}}_{p}^{n}$ acting as the ciphertext policy, and outputs the ciphertext *CT*_*M*_ consisting of

(16) \begin{eqnarray*}\begin{array}{@{}rl@{}} \displaystyle c{t}_{0} & ={g}_{1}^{\mathbi{As}} & \\ \displaystyle c{t}_{i} & ={g}_{1}^{({x}_{i}{\mathbi{U}}^{\top }+{\mathbi{X}}_{i}^{\top })\mathbi{As}}&\displaystyle \\ \displaystyle c{t}^{{^{\prime}}} & =M\cdot \hat {e}({g}_{1},{g}_{2})^{{\tau }^{\top }\mathbi{As}}, \end{array}\end{eqnarray*}

where $\tau ={\mathop{\sum }\nolimits }_{i=1}^{n}{\tau }_{i}$.

Lastly, the ciphertext *CT*_*M*_ is sent to the contract *SC*_*log*_ with the optional keyword *kw* that may ease the data retrieval process. The contract *SC*_*log*_ will emit this new uploading information to the subscribers.

### Download and decryption

All the encrypted metadata *CT*_*M*_ will be recorded sequentially. If the *DU* is interested in one of *DO*’s files, *DU* may subscribe to the event created by the contract *SC*_*log*_ to obtain the encrypted metadata *CT*_*M*_.

To decrypt the ciphertext *CT*_*M*_, *DU* computes

\begin{eqnarray*}\hat {e}(c{t}_{0},\prod _{j=1}^{l+1}{K}_{j})\cdot \hat {e}(\prod ct_{i}^{{v}_{i}},\mathbi{h})=\hat {e}({g}_{1},{g}_{2})^{{\tau }^{\top }As} \end{eqnarray*}

and tries to recover the message

(17) \begin{eqnarray*}c{t}^{{^{\prime}}}/\hat {e}({g}_{1},{g}_{2})^{{\tau }^{\top }As}={M}^{{^{\prime}}}\end{eqnarray*}

If *DU*’s attribute vector ***v*** satisfies the policy vector ***x*** selected by *DO*, *DU* can retrieve the AES key *AK* and file location *loc* from the metadata *M*. Finally, *DU* downloads the encrypted file *CT*_*F*_ from the IPFS based on the location *loc* and uses *AK* to decrypt *CT*_*F*_ to recover the original file *F*.

*Correctness.* Since $C{T}_{M}=(c{t}_{0},\{ c{t}_{i}\} _{i=1}^{n},c{t}^{{^{\prime}}})$, $&lcub; s{k}_{i,GID,\mathbi{C}}={&lcub; {K}_{j}&rcub; }_{j&isin; {\mathcal{R}}_{i}}&rcub; $ and *n* = *l* + 1, we can compute

(18) \begin{eqnarray*}\begin{array}{@{}rl@{}} \displaystyle & \hat {e}(c{t}_{0},\prod _{j=1}^{l+1}{K}_{j})\cdot \hat {e}(\prod _{i=1}^{n}c{t}_{i}^{{v}_{i}},\mathbi{H}(GID,\mathbi{C})) & \\ \displaystyle & =\hat {e}({g}_{1}^{\mathbi{As}},{g}_{2}^{\sum _{i=1}^{n}{\tau }_{i}-{v}_{i}{\mathbi{X}}_{i}h+{\mu }_{i}})&\displaystyle \\ \displaystyle & \cdot \hat {e}({g}_{1}^{\sum _{i=1}^{n}{v}_{i}({x}_{i}{\mathbi{U}}^{\top }+{\mathbi{X}}_{i}^{\top })\mathbi{As}},{g}_{2}^{\mathbi{h}})&\displaystyle \\ \displaystyle & =\hat {e}({g}_{1},{g}_{2})^{{\tau }^{\top }\mathbi{As}-\sum _{i=1}^{n}{v}_{i}{\mathbi{h}}^{\top }{\mathbi{X}}_{i}^{\top }\mathbi{As}}&\displaystyle \\ \displaystyle & \cdot \hat {e}({g}_{1},{g}_{2})^{{< x,v> h}^{\top }{U}^{\top }As+\sum _{i=1}^{n}{v}_{i}{\mathbi{h}}^{\top }{X}_{i}^{\top }As}&\displaystyle \\ \displaystyle & =\hat {e}({g}_{1},{g}_{2})^{{\tau }^{\top }\mathbi{As}}\cdot \hat {e}({g}_{1},{g}_{2})^{{< x,v> h}^{\top }{U}^{\top }As} \end{array}\end{eqnarray*}

If <***x,v*** >  = 0, we obtain $\hat {e}({g}_{1},{g}_{2})^{{\tau }^{\top }As}$ and can recover the message.

## Security Analysis

The security of the original scheme (Definition 5), while explored within the framework by Michalevsky & Joye (2018) and proven to be secure against selective adversaries in random oracle model (ROM), does not adequately capture certain real-world circumstances. Recognizing this limitation, we have devised a more flexible approach to model the security challenges more accurately.

This new model, defined in Definition 6, is derived from the original scheme but adapted to bridge the gap between theoretical models and the complexities of real-world applications. In Datta, Komargodski & Waters (2023), this notion has been defined as fully adaptive security, where the adversary can decide the set of authorities to corrupt at any time.

Definition 6 The scheme is secure against static corruption of authorities if the advantage in winning the following game is negligible.

1. **Setup phase:** $\mathcal{S}$ picks a random bit *b* ∈ {0, 1} and outputs the public parameters *PP*.
2. **Query phase:**
  1 $\mathcal{A}$ can adaptively decide the identity of each attribute authority, determining whether it is normal or corrupted, as well as the order in which each authority’s keys are generated or received by $\mathcal{S}$.
    • If the authority is normal, $\mathcal{A}$ requests the keys {*PK*} from $\mathcal{S}$, which are generated through the **Auth Setup** process.
    • If the authority is corrupted, $\mathcal{A}$ generates the keys {*PK*, *SK*} itself and provides {*PK*} to $\mathcal{S}$.
  2 **Finally**, $\mathcal{A}$ marks each corrupted authority and outputs the set of corrupt authorities *A*^∗^ based on the decisions made in the previous step.
3. **Challenge phase:**
  1 $\mathcal{A}$ outputs two policies ***x***_0_, ***x***_1_ and two equal-length messages *M*_0_, *M*_1_.
  2 $\mathcal{S}$ outputs a challenge ciphertext $CT\leftarrow _{}^{\text{$}}\mathbf{Encrypt}({\mathbi{x}}_{b},{M}_{b})$, where *b* = 0 or 1
4. **Query phase 2:** $\mathcal{A}$ request secret attribute keys {*sk*} for vectors ***v*** from $\mathcal{S}$, which are not orthogonal to ***x***_0_ or ***x***_1_.
5. **Guess:** $\mathcal{A}$ outputs a guess *b*′ and wins the game if *b*′ = *b*.

Contrasting with Michalevsky & Joye (2018) in the **Query Phase**, which requires the adversary $\mathcal{A}$ fix a set of corrupt authorities and provide their public parameters *PK* **prior** to $\mathcal{S}$ executes **AuthSetup** for the non-corrupt authorities as follows:

**Query phase:**

1. $\mathcal{A}$ **first** outputs the set of corrupt authorities *A*^∗^, generates authority keys {*PK*, *SK*}_*i*_ for every *AA*_*i*_ ∈ *A*^∗^, and provides {*PK*}_*i*_ to $\mathcal{S}$.
2. $\mathcal{A}$ **then** requests the remaining public keys {*PK*}_*j*_ generated from $\mathcal{S}$ for each non-corrupt authority *AA*_*j*_⁄ ∈ *A*^∗^.

This adaptation introduces an aspect of real-world unpredictability and tests the system’s robustness on a broader array of scenarios.

## Rogue-key attack analysis

First, we provide proof that the original scheme, as proposed by Michalevsky & Joye (2018) and outlined in Definition 5, is vulnerable to a rogue-key attack in the game (Definition 6). Following this, we discuss how our blockchain-based data governance mechanism effectively mitigates this vulnerability.

### Attack

We demonstrate that a corrupt authority can learn the key parts *K*_*l*+1_ corresponding to *v*_*l*+1_ ∈ ***v*** issued by trusted authority *AA*_trust_ and thus decrypt the ciphertext in Michalevsky and Joys’s scheme (Michalevsky & Joye, 2018), without having the required secret key *sk* satisfying the attached access policy. This is a typical attack, called a rogue-key attack, in which the adversary uses a public key, a function of honest users’ keys (Ristenpart & Yilek, 2007).

Proof First, an adversarial authority *AA*_*ad*_ makes one of the corrupted authorities hold until all the other attribute authorities *AA*_*i*_, *i* ≠ *ad*, *i* ∈ *n* publish their public keys, in accordance with the game (Definition 6).

\begin{eqnarray*}P{K}_{i}=({g}_{1}^{{\mathbi{X}}_{i}^{\top }\mathbi{A}},\hat {e}({g}_{1},{g}_{2})^{{\tau }_{i}^{\top }\mathbi{A}},y\coloneq {g}_{2}^{\sigma }) \end{eqnarray*}

and then calculates the public key as follows:

\begin{eqnarray*}{g}_{1}^{{\mathbi{X}}_{ad}^{\top }\mathbi{A}}\coloneq -\sum _{j=0,j\not = ad}^{n}{g}_{1}^{{\mathbi{X}}_{j}^{\top }\mathbi{A}} \end{eqnarray*}

\begin{eqnarray*}\hat {e}({g}_{1},{g}_{2})^{{\tau }_{ad}^{\top }\mathbi{A}},{y}_{ad}\coloneq {g}_{2}^{{\sigma }_{i}} \end{eqnarray*}

After receiving the challenge ciphtertext *CT*≔{*ct*_0_, *ct*_*i*_, *ct*′} from $\mathcal{S}$ in the **Challenge Phase**, an adversarial data user *DU*_*ad*_ creates an attribute vector ***v***′ = (0, 0, …, 0, 1) and requrests key parts *K*_*i*_ from all the attribute authorities *AA*_*i*_. As in the encryption phase, data owner *DO* will collect all the public keys *PK*_*i*_, *i* ∈ [*n* + 1] from each *AA*_*i*_ and outputs the cipher text *CT* consisting of

\begin{eqnarray*}c{t}_{0}={g}_{1}^{\mathbi{As}} \end{eqnarray*}

\begin{eqnarray*}c{t}_{i}={g}_{1}^{({x}_{i}{\mathbi{U}}^{\top }+{\mathbi{X}}_{i}^{\top })\mathbi{As}} \end{eqnarray*}

\begin{eqnarray*}c{t}^{{^{\prime}}}=m\cdot \hat {e}({g}_{1},{g}_{2})^{{\tau }^{\top }\mathbi{As}}, \end{eqnarray*}

where $\tau ={\mathop{\sum }\nolimits }_{i=1}^{n}{\tau }_{i}$. Third, given the ciphertest (*ct*_0_, {*ct*_*i*_}, *ct*′) and received secret keys {*K*_*i*_}, adversary *DU*_*ad*_ decrypts it as following:

\begin{eqnarray*}\hat {e}& (c{t}_{0},\prod _{j=1}^{l+1}{K}_{i})\cdot \hat {e}(\prod _{i=1}^{n}c{t}_{i}^{{v}_{i}},\mathbi{H}(GID,\mathbi{C}))\nonumber\\\displaystyle & =\hat {e}({g}_{1}^{\mathbi{As}},{g}_{2}^{\sum _{i=1}^{n}{\tau }_{i}-{v}_{i}{\mathbi{X}}_{i}\mathbi{h}+{\mu }_{i}})\nonumber\\\displaystyle & \cdot \hat {e}({g}_{1}^{\sum _{i=1}^{n}{v}_{i}({x}_{i}{\mathbi{U}}^{\top }+{\mathbi{X}}_{i}^{\top })\mathbi{As}},{g}_{2}^{\mathbi{h}})\nonumber\\\displaystyle & =\hat {e}({g}_{1},{g}_{2})^{{\tau }^{\top }\mathbi{As}-\sum _{i=1}^{n}{v}_{i}{\mathbi{h}}^{\top }{\mathbi{X}}_{i}^{\top }\mathbi{As}}\nonumber\\\displaystyle & \cdot \hat {e}({g}_{1},{g}_{2})^{< x,v> {\mathbi{h}}^{\top }{\mathbi{U}}^{\top }\mathbi{A}s+\sum _{i=1}^{n}{v}_{i}{\mathbi{h}}^{\top }{\mathbi{X}}_{i}^{\top }\mathbi{As}}\nonumber\\\displaystyle & =\hat {e}({g}_{1},{g}_{2})^{{\tau }^{\top }\mathbi{As}}\cdot \hat {e}({g}_{1},{g}_{2})^{{< x,v> h}^{\top }{\mathbi{U}}^{\top }\mathbi{As}} \end{eqnarray*}

Since *DU*_*ad*_’s attribute vector is ***v***′ = (0, 0, …, 0, 1), we have

(19) \begin{eqnarray*}\hat {e}({g}_{1},{g}_{2})^{{\tau }^{\top }\mathbi{As}}\cdot \hat {e}({g}_{1},{g}_{2}^{\mathbi{h}})^{{x}_{l+1}{\mathbi{U}}^{\top }\mathbi{As}}\end{eqnarray*}

In order to decrypt the ciphertext, we need to cancel out the last element in Eq. (19). Therefore, adversary $\mathcal{A}$ could use the published {*ct*_*i*_} to calculate attacking component *ω*.

\begin{eqnarray*}\omega \coloneq \prod g_{1}^{({x}_{i}{\mathbi{U}}^{\top }+{\mathbi{X}}_{i}^{\top })\mathbi{As}}\nonumber\\\displaystyle ={g}_{1}^{\sum {x}_{i}{\mathbi{U}}^{\top }\mathbi{As}}\cdot {g}_{1}^{\sum (\mathbi{X}_{i}^{\top }\mathbi{A})\mathbi{s}}. \end{eqnarray*}

Based on ${g}_{1}^{{\mathbi{X}}_{ad}^{\top }\mathbi{A}}\coloneq -{\mathop{\sum }\nolimits }_{j=0,j\not = ad}^{n}{g}_{1}^{{\mathbi{X}}_{j}^{\top }\mathbi{A}}$, we obtain $\omega ={g}_{1}^{{\mathop{\sum }\nolimits }_{i=1}^{n}{x}_{i}{\mathbi{X}}^{\top }\mathbi{As}}$ and can further cancel the last component in Eq. (19) as ${x}_{l+1}=-{\mathop{\sum }\nolimits }_{i=1}^{n}{x}_{i}$. In the end, the adversary recovers the message by computing

\begin{eqnarray*}c{t}^{{^{\prime}}}/\hat {e}({g}_{1},{g}_{2})^{{\tau }^{\top }\mathbi{As}}=m. \end{eqnarray*}

□

Based on the provided chosen message (*M*_0_, *M*_1_) in the **Challenge Phase**, $\mathcal{A}$ can easily output a correct guess *b*′ that *b*′ = *b* with high probability, which violates the game defined by Definition 6.

### Proof of security of our approach

In the absence of a certificate authority (CA), one way to mitigate this requires a Non-interactive Zero-knowledge (NIZK) proof in a decentralized manner. Since every *AA* needs to generate a commitment of secrets used for public key *PK* and then creates its proof of knowledge using NIZK ( Algorithm 2 ), which is built upon Schnorr Identification Protocol (Schnorr, 1990).

Proof Each attribute authority (*AA*) generate commitments to their secrets {***X***, *τ*, *σ*} and creates a NIZK of knowledge *π*, as described in **Authority Setup**. We thus just need to prove the NIZK satisfies the Knowledge of Coefficient assumption (Assumption 5) of the work Bowe, Gabizon & Miers (2017). Suppose an adversary $\mathcal{A}$ successfully crafts a proof *π*_*ad*,*s*_ = (*R*_*ad*,*s*_, *u*_*ad*,*s*_) for a secret $s={\mathop{\sum }\nolimits }_{i=1,i\not = ad}^{n-1}{\mathbi{X}}_{i}^{\top }\mathbi{A}$ for a secret *s* without knowing *s*, which implies that given *s-pair* ***rp***_*s*_ = (*A*, *B*) and string *h*, the probability of finding a valid *u* such that *u*⋅*A* = *R* + *c*⋅*B* would be **non-negligible**. Consiering another *s-pair* ${\mathbi{rp}}_{\sigma }^{2}=\{ {g}_{2},{g}_{2}^{\sigma }\} $, $\mathcal{A}$ computes ${R}^{{^{\prime}}}\leftarrow {g}_{1}^{{\alpha }^{{^{\prime}}}}$ and ${c}^{{^{\prime}}}\leftarrow _{}^{}\mathbf{Hash}({R}^{{^{\prime}}}{|}{|}h)$ as in Definition 2 based on the randomly chosen value *α*′. Assigning *r*≔*g*_2_ and $y\coloneq ({g}_{2}^{\sigma })^{{c}^{{^{\prime}}}}$ in the **SameRatio**((*g*_1_, *x*), (*r*, *y*)), which implies that $x={g}_{1}^{\sigma \cdot {c}^{{^{\prime}}}}$, if $\mathcal{A}$ can find *u*′ such that *u*′ = *α*′ + *c*′⋅*s*, this would satisfy the **SameRatio** ((*g*_1_, *x*), (*r*, *y*)) condition without knowing *σ*⋅*c*′, violating the Assumption 5. Thus, $\mathcal{A}$ cannot win the game, and our scheme is secure against rogue-key attacks.□

### Inferring the secret vector in ciphertext

With its further study of the Decentralized Policy-hiding ABE scheme (Michalevsky & Joye, 2018), we also identified a potential risk associated with generating public parameters during **Setup**. The details of the risk and proof of security are described in the following sections.

#### Vulnerability

As defined in Definition 5, a trusted third-party (TTP) or an attribute authority (*AA*) needs to pick a set of random numbers ${a}_{1},{a}_{2},\ldots ,{a}_{k}\leftarrow _{}^{\text{$}}{\mathbb{Z}}_{p}^{\ast }$, and then generate a random matrix

$\mathbi{A}= \left( {\scriptsize \begin{array}{@{}cccc@{}} \displaystyle {a}_{1}&\displaystyle 0&\displaystyle &\displaystyle 0\\ \displaystyle 0&\displaystyle {a}_{2}&\displaystyle &\displaystyle 0\\ \displaystyle \vdots &\displaystyle \ddots &\displaystyle &\displaystyle \\ \displaystyle 0&\displaystyle 0&\displaystyle \cdots &\displaystyle {a}_{k}\\ \displaystyle 1&\displaystyle 1&\displaystyle \cdots &\displaystyle 1\\ \displaystyle \end{array}} \right) \in {\mathbb{Z}}_{p}^{(k+1)\times k}$ with ${\mathbi{a}}^{\perp }= \left( {\scriptsize \begin{array}{@{}c@{}} \displaystyle {a}_{1}^{-1}\\ \displaystyle {a}_{2}^{-1}\\ \displaystyle \vdots \\ \displaystyle {a}_{k}^{-1}\\ \displaystyle -1\\ \displaystyle \end{array}} \right) \in {\mathbb{Z}}_{p}^{(k+1)}$, which ***A***^⊺^***a***^⊥^ = 0 during **Setup**.

Proof If the above generation is conducted by a PPT adversary $\mathcal{A}$, or if the sensitive information ***a***^⊥^ is exposed, $\mathcal{A}$ can infer the value of ${g}_{1}^{\mathbi{s}}$ from any published ciphertext *CT*. As we know, to generate a ciphertext *CT*, data owner (*DO*) firstly samples a random vector ***s*** = (*s*_1_, *s*_2_, $\ldots ,{s}_{k})\in {\mathbb{Z}}_{p}^{k}$, then creates a policy vector $\mathbi{x}=({x}_{1},{x}_{2},\ldots ,{x}_{n-1},{x}_{n})\in {\mathbb{Z}}_{p}^{n}$ acting as the ciphertext policy, and finally outputs the ciphertext *CT* consisting of

\begin{eqnarray*}c{t}_{0}={g}_{1}^{\mathbi{As}} \end{eqnarray*}

\begin{eqnarray*}c{t}_{i}={g}_{1}^{({x}_{i}{\mathbi{U}}^{\top }+{\mathbi{X}}_{i}^{\top })\mathbi{As}} \end{eqnarray*}

\begin{eqnarray*}c{t}^{{^{\prime}}}=M\cdot \hat {e}({g}_{1},{g}_{2})^{{\tau }^{\top }\mathbi{As}} \end{eqnarray*}

where $\tau ={\mathop{\sum }\nolimits }_{i=1}^{n}{\tau }_{i}$ and $\hat {e}({g}_{1},{g}_{2})^{{\tau }_{i}^{\top }}$ collected from published public keys *PK*_*i*_.

Since

\begin{eqnarray*}\mathbi{As}= \left( \begin{array}{@{}c@{}} \displaystyle {a}_{1}{s}_{1}\\ \displaystyle {a}_{2}{s}_{2}\\ \displaystyle \vdots \\ \displaystyle {a}_{k}{s}_{k}\\ \displaystyle {s}_{1}+{s}_{2}+\cdots +{s}_{k}\\ \displaystyle \end{array} \right) \end{eqnarray*}

$\mathcal{A}$ can use the result $c{t}_{0}={g}_{1}^{\mathbi{As}}$ and known ***a*** to calculate:

\begin{eqnarray*}({g}_{1}^{\mathbi{As}})^{\mathbi{a}}={g}_{1}^{\mathbi{Asa}} \end{eqnarray*}

For the exponent ***Asa***, we have

\begin{eqnarray*}\mathbi{Asa}& = \left( \begin{array}{@{}c@{}} \displaystyle {a}_{1}{s}_{1}\\ \displaystyle {a}_{2}{s}_{2}\\ \displaystyle \vdots \\ \displaystyle {a}_{k}{s}_{k}\\ \displaystyle {s}_{1}+{s}_{2}+\cdots +{s}_{k}\\ \displaystyle \end{array} \right) \cdot ({a}_{1}^{-1},{a}_{2}^{-1},\ldots ,{a}_{k}^{-1},-1)\nonumber\\\displaystyle & = \left( \begin{array}{@{}ccccc@{}} \displaystyle {s}_{1}&\displaystyle {a}_{1}{a}_{2}^{-1}{s}_{1}&\displaystyle \ldots &\displaystyle {a}_{1}{a}_{k}^{-1}{s}_{1}&\displaystyle -{a}_{1}{s}_{1}\\ \displaystyle {a}_{1}^{-1}{a}_{2}{s}_{2}&\displaystyle {s}_{2}&\displaystyle \ldots &\displaystyle {a}_{2}{a}_{k}^{-1}{s}_{2}&\displaystyle -{a}_{2}{s}_{2}\\ \displaystyle \vdots &\displaystyle \ddots &\displaystyle &\displaystyle \\ \displaystyle {a}_{1}^{-1}{a}_{k}{s}_{k}&\displaystyle {a}_{2}^{-1}{a}_{k}{s}_{k}&\displaystyle \ldots &\displaystyle {s}_{k}&\displaystyle -{a}_{k}{s}_{k}\\ \displaystyle {a}_{1}^{-1}\sum s&\displaystyle {a}_{2}^{-1}\sum s&\displaystyle \cdots &\displaystyle {a}_{k}^{-1}\sum s&\displaystyle -\sum s\\ \displaystyle \end{array} \right) \end{eqnarray*}

The *i*th elements from exponents ***As*** and ***a*** partially cancel each other out, and the remaining element *s*_*i*_ is the targeting exponent. Therefore, adversary $\mathcal{A}$ might extrapolate ${g}_{1}^{\mathbi{s}}$ from the published ciphertext $c{t}_{0}={g}_{1}^{\mathbi{Asa}}$ with the knowledge of ***a***^⊥^.□

#### Proof of security with our approach

One potential mitigation of the inferring attack is to generate a composite public parameter *PP* by multiple *AA*, such that neither of those individual *AA*s knows the composite

\begin{eqnarray*}{g}_{1}^{\mathbi{A}}=powerMulti(powerMulti(\ldots (\nonumber\\\displaystyle powerMulti(powerMulti({g}_{1}^{{\mathbi{A}}_{1}},{\mathbi{A}}_{2}),{\mathbi{A}}_{3})\ldots ,{\mathbi{A}}_{n}), \end{eqnarray*}

and no participant learns the secrets of others unless one colludes with every other participant under Assumption 5.

Another potential security vulnerability arises from inconsistencies in the published sets $({V}_{i},{\theta }_{{V}_{i}},{V}_{i}^{{^{\prime}}})$ or $({W}_{i},{\theta }_{{W}_{i}},{W}_{i}^{{^{\prime}}})$ that, *i.e.,* an adversary *AA* with index *i* can strategically choose different $({\mathbi{A}}_{i},{\mathbi{A}}_{i}^{{^{\prime}}})$ values to compute

\begin{eqnarray*}{V}_{i}& \coloneq powerMulti({V}_{i-1},{\mathbi{A}}_{i}^{{^{\prime}}}) \end{eqnarray*}

\begin{eqnarray*}{V}_{i}^{{^{\prime}}}& \coloneq powerMulti({V}_{i-1}^{{^{\prime}}},{\alpha }_{{\mathbi{ A}}_{i}}{\mathbi{A}}_{i}), \end{eqnarray*}

results in the final composite becoming invalid for further operations. However, given that each *AA* has committed and disclosed a set of group elements

\begin{eqnarray*}e=\{ \mathbi{A},\mathbi{U},{\alpha }_{\mathbi{A}},{\alpha }_{\mathbi{U}},{\alpha }_{\mathbi{A}}\cdot \mathbi{A},{\alpha }_{\mathbi{U}}\cdot \mathbi{U}\} \end{eqnarray*}

in both 𝔾_1_ and 𝔾_2_, as described in “Commit and Reveal”, it becomes possible to ascertain that each *AA*’s $({V}_{i},{\theta }_{{V}_{i}},{V}_{i}^{{^{\prime}}})$ is a proper multiple of previous published components:

\begin{eqnarray*}\hat {e}({V}_{i},{g}_{2})& =\hat {e}({V}_{i-1},{g}_{2}^{{\mathbi{A}}_{i}}) \end{eqnarray*}

\begin{eqnarray*}\hat {e}({\theta }_{{V}_{i}},{g}_{2})& =\hat {e}({\theta }_{{V}_{i-1}},{g}_{2}^{{\alpha }_{i}}) \end{eqnarray*}

\begin{eqnarray*}\hat {e}({V}_{i}^{{^{\prime}}},{g}_{2})& =\hat {e}({V}_{i-1}^{{^{\prime}}},{g}_{2}^{{\alpha }_{{\mathbi{A}}_{i}}{\mathbi{A}}_{i}}) \end{eqnarray*}

Even if all the other participants have collaborated, this is still a robust and sufficient setup scheme, even if only one participant is honest and does not reveal the secrets. Hence, the greater the number of unrelated participants in a trusted setup, the less likely the possibility of invalid and unusable public parameter *PP*_*ABE*_ (Wilcox, 2016).

### Receiver privacy

According to the Definition 5, the receiver must provide its attribute vector ***v*** to each attribute authority *AA* from which a key is requested. In consequence, *AA* learns not only if the user possesses the attribute that *AA* owns but also all of the user’s other attributes. This appears to violate the privacy of the user in a decentralized setting.

Therefore, Michalevsky and Joye propose an enhancement that provides additional privacy protection for the attribute vector ***v*** in their work Michalevsky & Joye (2018), which uses it to hide the set of receiver attributes ***v*** from authorities. This technique is called *position-binding* Vector Commitments, introduced by Catalano & Fiore (2013). Our access control system is based on the work Catalano & Fiore (2013), but it has been slightly modified to work with asymmetric pairings $\hat {e}:{\mathbb{G}}_{1}\times {\mathbb{G}}_{2}\rightarrow {\mathbb{G}}_{T}$.

Since hiding is not a critical property and can be easily achieved in the realization of vector commitments (Catalano & Fiore, 2013), only the proof of position binding is provided below:

Proof Suppose an efficient adversary $\mathcal{A}$ can produce two valid openings *op* to different messages (*m*, *m*′) at the same position *i*. In that case, we can build an algorithm $\mathcal{B}$ that uses $\mathcal{A}$ to break the Square Computational Diffie-Hellman assumption. For a sequence of messages *m*_1_, *m*_2_, …, *m*_*n*_ and public parameter *pp* = (*g*_1_, *g*_2_, {*o*_*i*_}_*i*∈[*n*]_, {*o*_*i*,*j*_}_*i*,*j*∈[*n*],*i*≠*j*_), the vector commitment is $\mathbi{C}={o}_{1}^{{m}_{1}}{o}_{2}^{{m}_{2}}\cdot {o}_{n}^{{m}_{n}}$ and the opening for position *i* is $o{p}_{i}=({\mathop{\prod }\nolimits }_{j=1,j\not = i}^{n}{o}_{j}^{{m}_{j}})^{{z}_{i}}$. The efficient algorithm $\mathcal{B}$ takes as input a tuple $({g}_{1},{g}_{1}^{r},{g}_{2}^{r})$ and aims to compute ${g}_{1}^{{r}^{2}}$ to break Assumption 4. First, $\mathcal{B}$ selects a random position $i\leftarrow _{}^{\text{$}}[n]$ on which adversary $\mathcal{A}$ will break the position binding. Next, $\mathcal{B}$ chooses ${z}_{j}\leftarrow _{}^{\text{$}}{\mathbb{Z}}_{p}^{\ast }$ where ∀*j* ∈ [*n*], *j* ≠ *i* and then computes:

\begin{eqnarray*}\forall j\in [n]\setminus i:{o}_{j}={g}_{1}^{{z}_{j}},{o}_{i,j}=({g}_{1}^{r})^{{z}_{j}},{o}_{i}={g}_{1}^{r} \end{eqnarray*}

\begin{eqnarray*}\forall k,j\in [n]\setminus i,k\not = j:{o}_{k,j}={g}_{1}^{{z}_{k}{z}_{j}} \end{eqnarray*}

Second, $\mathcal{B}$ sets *pp* = (*g*_1_, *g*_2_, {*o*_*i*_}_*i*∈[*n*]_, {*o*_*i*,*j*_}_*i*,*j*∈[*n*],*i*≠*j*_) and runs $\mathcal{A}(pp)$, that outputs a tuple (***C***, *m*, *m*′, *j*, 

$o{p}_{j},o{p}_{j}^{{^{\prime}}})$ such that *m* ≠ *m*′ and both *op*_*j*_ and $o{p}_{j}^{{^{\prime}}}$ are valid.

Finally, $\mathcal{B}$ computes

\begin{eqnarray*}{g}_{1}^{{r}^{2}}=(o{p}_{i}^{{^{\prime}}}/o{p}_{i})^{({m}_{i}-{m}_{i}^{{^{\prime}}})^{-1}} \end{eqnarray*}

Since openings verify the two messages $({m}_{i},{m}_{i}^{{^{\prime}}})$ correctly at position *i*, then it holds:

\begin{eqnarray*}\hat {e}(\mathbi{C},{g}_{2}^{r})=\hat {e}({o}_{i}^{{m}_{i}},{g}_{2}^{r})\hat {e}(o{p}_{i},{g}_{2})=\hat {e}({o}_{i}^{{m}_{i}^{{^{\prime}}}},{g}_{2}^{r})\hat {e}(o{p}_{i}^{{^{\prime}}},{g}_{2}) \end{eqnarray*}

which means that

\begin{eqnarray*}\hat {e}({o}_{i},{g}_{2}^{r})^{{m}_{i}-{m}_{i}^{{^{\prime}}}}=\hat {e}(o{p}_{i}^{{^{\prime}}}/o{p}_{i},{g}_{2}) \end{eqnarray*}

Since ${o}_{i}={g}_{1}^{r},o{p}_{i}^{{^{\prime}}}/o{p}_{i}=({g}_{1}^{{r}^{2}})^{{m}_{i}-{m}_{i}^{{^{\prime}}}}$, we have:

\begin{eqnarray*}\hat {e}({o}_{i},{g}_{2}^{r})^{{m}_{i}-{m}_{i}^{{^{\prime}}}}=\hat {e}({g}_{1},{g}_{2})^{{r}^{2}({m}_{i}-{m}_{i}^{{^{\prime}}})} \end{eqnarray*}

\begin{eqnarray*}\hat {e}(o{p}_{i}^{{^{\prime}}}/o{p}_{i},{g}_{2})=\hat {e}(({g}_{1}^{{r}^{2}})^{{m}_{i}-{m}_{i}^{{^{\prime}}}},{g}_{2})=\hat {e}({g}_{1},{g}_{2})^{{r}^{2}({m}_{i}-{m}_{i}^{{^{\prime}}})}, \end{eqnarray*}

which justifies the correctness of $\mathcal{B}$’s output. Therefore, if the Square Computational Diffie-Hellan assumption holds, the scheme satisfies the position-binding property.□

### Other security requirements

#### Policy-hiding

Policy-hiding means that the ciphertext policy is hidden from inspection. In our approach, we achieve a weaker concept known as weakly attribute-hiding, which ensures that the policy remains unknown to all parties except those who can decrypt the ciphertext. Our access control system is constructed on top of the decentralized inner-product predicate encryption scheme in Michalevsky & Joye (2018). It provides detailed proof that, in the existence of corrupted authorities, the advantage of a PPT adversary $\mathcal{A}$ in winning a sequence of games is negligible in the security parameter *λ*.

#### Trustability

Most existing solutions require an intermediary entity to ensure reliable and secure data management, resulting in expensive costs to prevent a single point of failure and privacy leakage. To overcome these obstacles, our approach employs the characteristics of blockchain distribution, decentralization, transparency, and immutability. By publishing the encrypted metadata, which consists of the AES key *AK* and file location *loc*, to the blockchain, we can maintain the integrity of access control management without requiring any intermediaries.

#### Traceability

Our system can track and validate access control data on the blockchain. Any activities, including setup, registration, key generation, encryption, and data uploading, are recorded as immutable transactions.

## Performance Analysis

In this section, we present a comparative analysis of our proposed Blockchain-enabled data governance system against existing related works Wang, Zhang & Zhang (2018), Qin et al. (2021), Nishide, Yoneyama & Ohta (2008), Gao et al. (2020), Cui et al. (2018), Zhang, Zheng & Deng (2018), Yang et al. (2018), Zhao et al. (2022), Tseng & Gao (2022) in terms of performance and security. In Table 5, we position these aforementioned works that are closely related to our work, providing comprehensive comparisons in the bilinear mapping group, security model, and time complexity of **Auth Setup**, **Encryption**, and **Decryption**.

**Table 5:** Summary of system using attribute-based encryption.

**Approach**	**Group**	**Security**	**Auth setup**	**Encryption**	**Decryption**
Wang, Zhang & Zhang (2018)	Prime	S-Rom	1 pair + 2 exp	3 exp	2 pair
Cui et al. (2018)	Prime	S-STM	(16*I* + 3) exp + (12*I* + 9) pm	(8*I* + 2)exp	(6*I* + 1)pair + *I*exp
Zhang, Zheng & Deng (2018)	Composite	F-STM	5*I* exp + 6*I* pm	(6*I* + 4)exp^*^	(*I* + 2)pair + 2*I*exp^*^
Yang et al. (2018)	Composite	F-ROM	1 pair + 2 exp 2 pm	(7*I* + 1)exp	2*I*pair + 4*I*exp
Gao et al. (2020)	Composite	F-STM	1 pair + 2 exp + 2 pm	(*l* + 2)exp	(*l* + 1)pair
Qin et al. (2021)	Composite	F-Rom	1 pair + (2*n* + 2)exp + 2*n* pm	(5*I* + 1) exp+pair	2*I*pair+ *I*exp
Zhao et al. (2022)	Prime	S-STM	2 exp	5*I*pair + 4exp + 2pm	2*I*pair + 3*I*exp
Tseng & Gao (2022)	Prime	S-ROM	1 pair + (*l* + 2) exp	(*l* + 2)exp + (*ln* + 2*n* + *l* + 1)pm	2 pair + (*l* − 1) exp + *l*(*n* + 1) pm
This work	Prime	F-ROM^**^	2pair + 23exp + 16 pm	(2*n* + 1)exp	2 pair + 2*n* exp

**Notes.**

* Several works did not present the complexity information. Therefore, we have either extrapolated the complexity on our own or referenced results presented in the survey (Zhang et al., 2020b).

** This is not entirely accurate. We primarily address the rogue-key attack under the fully adaptive security framework. Thus, the actual security of our scheme is slightly stronger than selective security but does not fully achieve fully adaptive security.

It is important to note that real execution times, a critical aspect of performance evaluation, are often inconsistently reported in the literature. Among the works previously discussed, only (Gao et al., 2020; Cui et al., 2018; Zhang, Zheng & Deng, 2018; Zhao et al., 2022; Tseng & Gao, 2022) provide real execution times, but these are based on diverse platforms. Furthermore, the lack of a shared code base for these works precludes the replication of experiments to verify and compare execution times in a uniform environment.

As a result, we have selected a few works (Michalevsky & Joye, 2018; Tseng & Gao, 2022) for implementation. These works were chosen because both are IPPE-based schemes, and Tseng and Gao claimed that their work outperforms MJ’s scheme, upon which our construction is built, in the four algorithms: **Setup**, **Auth Setup**, **Encryption**, and **Decryption**.

It is important to note that we did not include the trusted setup in the comparison for the following reasons:

1. A malicious central authority is not the main concern of this work.
2. Traffic delays, such as transactions not being mined, are quite common in blockchain networks. Therefore, the complexity cost of the **trusted setup** may not accurately represent real-time execution. In contrast, proof generation and verification in **Auth Setup** for the attribute secret keys can be done offline, which is not impacted by network delays.

### Asymptotic comparisons

The notations used are: *exp* for exponentiation operation, *pair* for bilinear pair operation, *pm* for point multiplication operation, and *I* for access policy complexity for LSSS.

1. Group: There are two types of groups used in CP-ABE: prime-order and composite-order groups. It is noted that the design of prime-order CP-ABE is more efficient than composite-order CP-ABE but is more challenging to achieve full security.
2. Security model: Standard model (STM) and random oracle model (ROM) are two typical types of security models considered in the CP-ABE scheme. And adversaries are categorized into selective adversaries and adaptive adversaries. If a CP-ABE scheme is secure against adaptive adversaries under the standard model, we denote this scheme as F-STM achieving full security. Likewise, S-ROM represents a CP-ABE scheme robust against selective adversaries under the random oracle model, and F-ROM if it is secure against adaptive adversaries under the random oracle model.
3. Computation cost: This assessment considers both the encryption and decryption costs in terms of their complexity, measured in terms of certain standard (cryptographic) operations. The following notations are used:
  • *exp*: exponentiation operation
  • *pair*: bilinear pair operation
  • *pm*: point multiplication operation
  • *I*: the access policy complexity for LSSS
  • *l*: the size of attributes
  • *n*: the size of attribute authorities

In terms of computational costs, bilinear pairing (pair) is the most expensive operation compared to exponentiation (exp) and point multiplication (pm), and the LSSS is a special matrix whose rows are labeled by attributes such that the cost of *I* might be similar as *n* or *l*. Note that, each attribute authority is responsible for at least 1 attribute, which means that *n* ≤ *l*. Consequently, our scheme is superior to most of these works with respect to encryption and decryption cost, as shown in Table 5, but it is constrained in terms of the conjunction access policy.

### Experimental result

In this section, we present the experimental results of two IPPE-based schemes (Michalevsky & Joye, 2018; Tseng & Gao, 2022) and our enhanced construction, implemented in Python. We analyze the execution time in terms of all five algorithms defined for IPPE (Definition 5). The experiments were conducted on a platform featuring an i5-13600KF 20-Core processor with 128GB of RAM, running Ubuntu version 22.04.1 LTS. These implementations were developed using Python and the Charm-Crypto@0.50 cryptography library (Akinyele et al., 2013), utilizing the ‘MNT-159’ elliptic curve for asymmetric bilinear mapping and the ‘SS512’ elliptic curve for symmetric bilinear mapping. According to Cui et al. (2018), both curves provide an 80-bit security level. We set *k* = 2 as a standard value for the security parameter, which is widely adopted in similar works.

In our performance evaluation, we use the abbreviations MJ18, TG22, and Enhd to represent the three schemes from Michalevsky & Joye (2018), Tseng & Gao (2022), and our proposed enhanced IPPE-based scheme, respectively, as depicted in the following figures.

**Figure 2:**
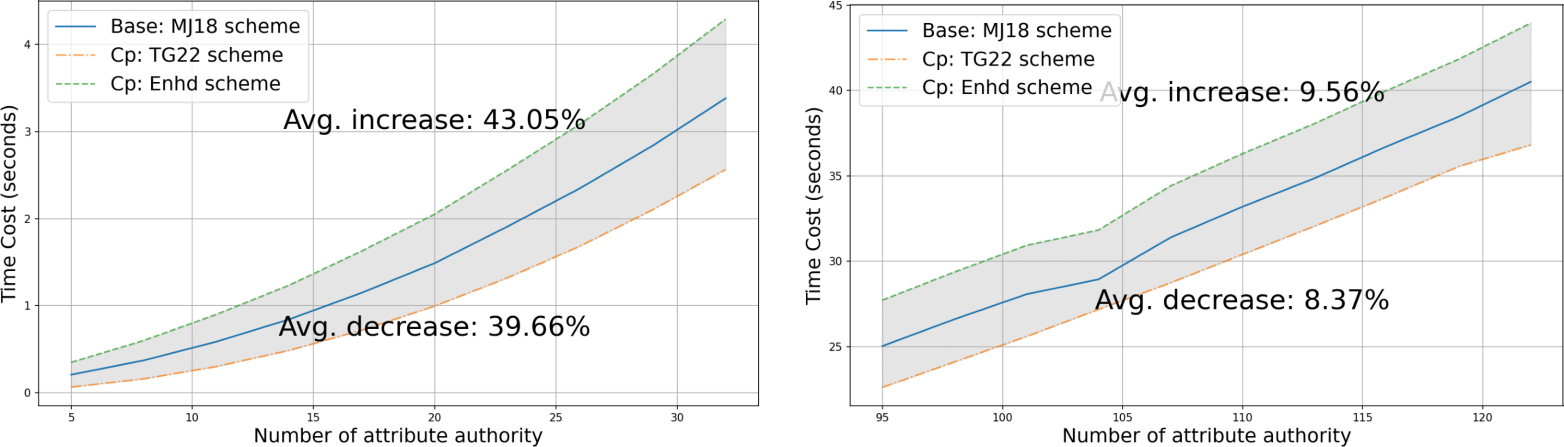
Comparision of total cost of schemes with varied numbers of attribute authorities.

Using Michalevsky and Joye’s work (Michalevsky & Joye, 2018) as the baseline (represented by the blue line in Fig. 2), we observe that our enhanced construction requires significantly more computation time, as indicated by the green dashed line. In contrast, Tseng & Gao (2022), depicted by the orange dash-dotted line, outperforms the baseline in efficiency. Meanwhile, we note that as the number of attribute authorities increases, the performance differences—both in terms of average increase and decrease—shrink from around 40%, as seen in Fig. 2A to 10%, as shown in Fig. 2B. We note that these relative computational cost overheads align with our expectations qualitatively, specifically, the enhanced security achieved with our approach incurs more computation.

**Figure 3:**
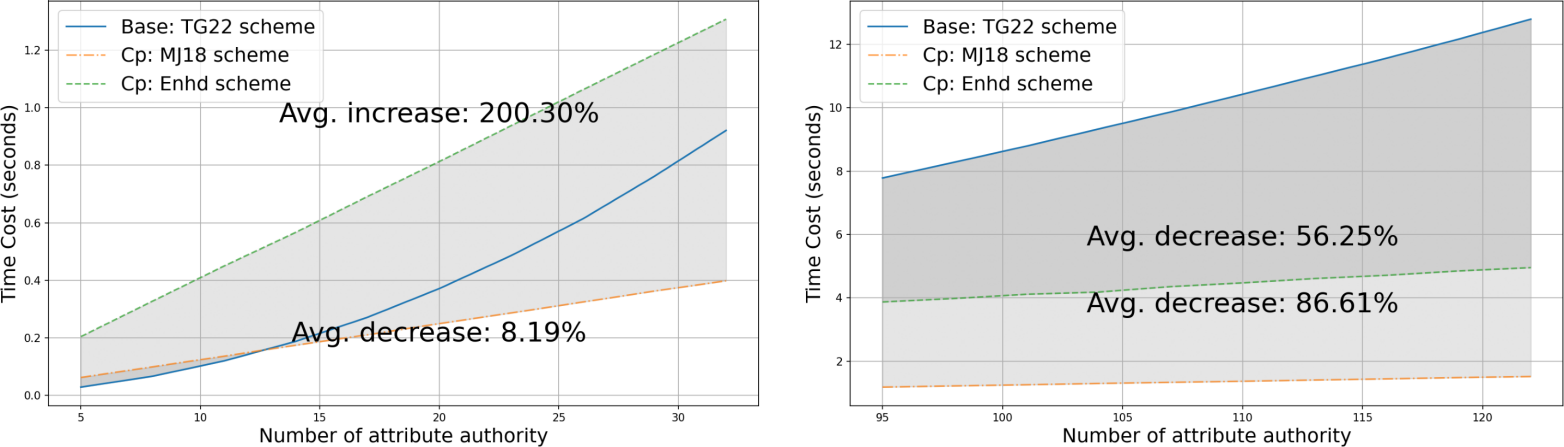
Comparison of auth setup with varied numbers of attribute authorities.

To further investigate the factors contributing to these performance differences quantitatively, we analyzed the time cost of each algorithm, focusing initially on the **Auth Setup** phase in Fig. 3.

Instead of using the work of Michalevsky & Joye (2018) as the baseline for total cost comparison, we have selected Tseng & Gao (2022) as the baseline for evaluating the performance of the **Auth Setup** algorithm. Surprisingly, although the scheme of Tseng & Gao (2022) scheme demonstrated efficiency in previous comparisons, its performance in the **Auth Setup** phase (represented by the blue line in Fig. 3) is not superior to Michalevsky & Joye (2018) (indicated by the orange dash-dotted line) even when the number of attribute authorities is small, as shown in Fig. 3A. Additionally, the performance gap between our proposed scheme (depicted by the green dashed line) and the scheme of Tseng & Gao (2022) narrows as the number of attribute authorities increases. When *n* exceeds 95, our enhanced scheme, which incorporates NIZK proofs to defend against rogue-key attacks, outperforms the scheme of Tseng & Gao (2022), as illustrated in Fig. 3B.

**Figure 4:**
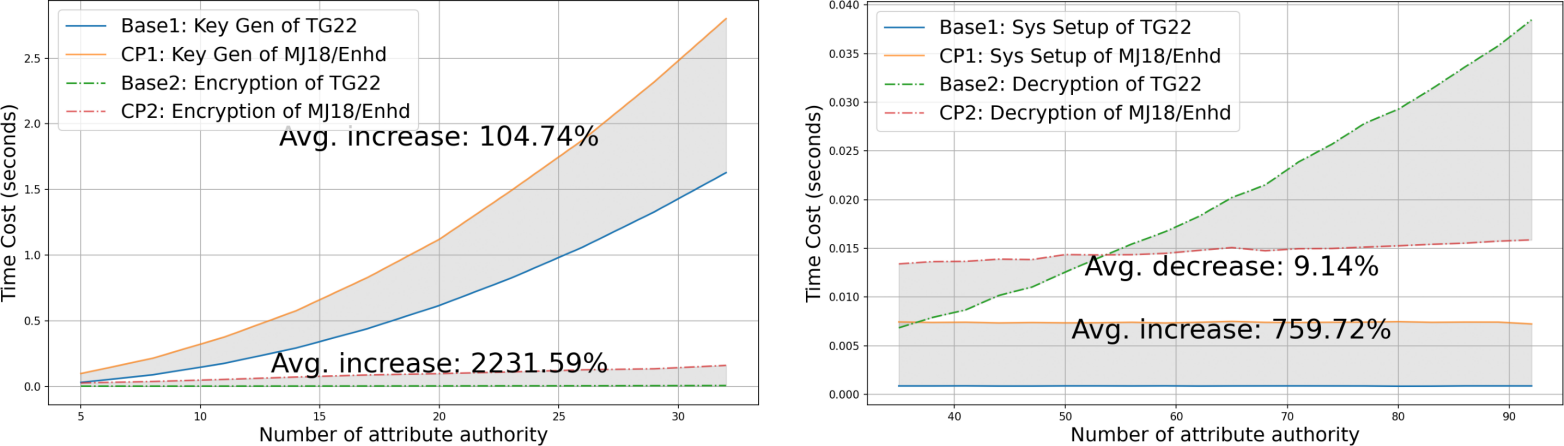
Comparison of other algorithms with varied numbers of attribute authorities.

Since our enhanced scheme only modifies the **Auth Setup**, the performance results of the other algorithms—**Sys Setup**, **Key Gen**, **Encryption**, and **Decryption**—are identical to those in Michalevsky & Joye (2018). The comparison among the three schemes is summarized in Fig. 4.

As shown in Figs. 4A and 4B, the **Encryption** and **Decryption** algorithms, all schemes (with the scheme of Tseng & Gao (2022) represented by the green dash-dotted line, Michalevsky & Joye (2018) and our enhanced scheme both by the red dash-dotted line) exhibit linear growth as the number of attribute authorities increases. Specifically, the **Decryption** algorithm in the work of Tseng & Gao (2022) increases at a faster rate than our enhanced scheme, surpassing our scheme’s time cost when the number of attribute authorities *n* reaches 53. For the **Key Gen** algorithms, all three schemes(with TG’s scheme represented by the blue line, MJ’s work, and ours both by the orange line) exhibit exponential growth; however, the performance gap widens as the number of authorities increases. In contrast, the **Sys Setup** algorithm remains stable and is unaffected by the number of attribute authorities, maintaining consistent performance even as *n* increases from 35 to 95.

In summary, although our proposed scheme with Non-Interactive Zero-Knowledge (NIZK) proofs to defend against rogue-key attacks performs better in the **Auth Setup** and **Decryption** algorithms compared to the scheme introduced in Tseng & Gao (2022), the poor performance of the inherited **Sys Setup**, **Encryption**, and especially **Key Generation** algorithms from Michalevsky & Joye (2018) causes our scheme to require additional time in the total cost, as shown in Fig. 2. However, as illustrated in Figs. 3B and 4B, the gap between our scheme and the work of Tseng & Gao (2022) narrows as the number of attribute authorities *n* increases, and eventually our scheme outperforms theirs. This indicates that the relative cost of the additional security measures in our scheme diminishes with a larger number of attribute authorities, suggesting that our scheme is suitable for scenarios involving a large number of attribute authorities, while, when the number of authorities is small, our approach is also practical in terms of the absolute cost even though it is relatively costlier than the other schemes.

## Concluding Remarks

Securing cloud data access and protecting identity privacy are legitimate concerns for many use cases, which this work addresses with a blockchain-based data governance system that is secure and privacy-preserving. A combination of attribute-based encryption (ABE) and the Advanced Encryption Standard (AES) makes the system efficient and responsive to real-world conditions. Our data-sharing system can handle multi-authority scenarios while protecting identity privacy and hiding ABE’s policy against rogue-key attacks under **fully adaptive corruption**.

However, because our system is built on top of Michalevsky & Joye (2018) and Bowe, Gabizon & Green (2018), it has inevitably inherited a few drawbacks: First, it only supports fixed-size attributes and authorities, which means that any changes to these may necessitate requesting a new system setup or a new key for *DU* from all authorities. Second, because each *AA*’s public key must be shared with others for the computation of masking terms *μ*_*i*_, the system requires coordination among authorities during the setup phase. Third, the implementation of a trusted setup to protect against rogue-key attacks adds complexity to the setup process, making it less suitable for scenarios where authorities frequently join or leave. Addressing these limitations is part of our planned future work.

Our proposed system addresses the key challenges of decentralization, practicality, and security. Specifically, it provides resilience against rogue-key attacks, considers dynamic setups through fully adaptive security, and mitigates the potential risk of information leakage caused by inference attacks. Considering the immediacy of the more fundamental security needs, the current work does not explore further common real-world desirable functionalities, such as interoperability, enabling data-sharing across different cloud environments, scalability, particularly in handling large-scale data and high-concurrency access, and other desired features like trustworthy and privacy-preserving keyword search schemes in encrypted decentralized storage. Furthermore, we aim to extend our research to address specific security compliance requirements in cloud environments, such as data governance, data sovereignty, and cross-regional data transfer. Addressing these aspects would significantly enhance the applicability of our system in real-world scenarios.

Thus, our future work will focus on addressing these shortcomings while further exploring ABE-based data-sharing systems in terms of decentralization, flexibility, interoperability, scalability, and security.

## Additional Information and Declarations

### Data Availability

The following information was supplied regarding data availability: The code is available at Zenodo: Zhang, J. (2024). Performance comparison in three IPPE-based schemes. Zenodo. https://doi.org/10.5281/zenodo.13950139.
